# Enhancing the Nutritional Quality of Major Food Crops Through Conventional and Genomics-Assisted Breeding

**DOI:** 10.3389/fnut.2020.533453

**Published:** 2020-11-26

**Authors:** Kiran B. Gaikwad, Sushma Rani, Manjeet Kumar, Vikas Gupta, Prashanth H. Babu, Naresh Kumar Bainsla, Rajbir Yadav

**Affiliations:** ^1^Division of Genetics, Indian Council of Agricultural Research (ICAR)-Indian Agricultural Research Institute, New Delhi, India; ^2^Indian Council of Agricultural Research (ICAR)-National Institute for Plant Biotechnology, New Delhi, India; ^3^Division of Genetics, Indian Council of Agricultural Research (ICAR)-Indian Institute of Wheat and Barley Research, Karnal, India

**Keywords:** nutritional quality, genomics assisted breeding, QTLs, food crops, malnutrition, genome editing, CRISPR/Cas9, plant breeding

## Abstract

Nutritional stress is making over two billion world population malnourished. Either our commercially cultivated varieties of cereals, pulses, and oilseed crops are deficient in essential nutrients or the soils in which these crops grow are becoming devoid of minerals. Unfortunately, our major food crops are poor sources of micronutrients required for normal human growth. To overcome the problem of nutritional deficiency, greater emphasis should be laid on the identification of genes/quantitative trait loci (QTLs) pertaining to essential nutrients and their successful deployment in elite breeding lines through marker-assisted breeding. The manuscript deals with information on identified QTLs for protein content, vitamins, macronutrients, micro-nutrients, minerals, oil content, and essential amino acids in major food crops. These QTLs can be utilized in the development of nutrient-rich crop varieties. Genome editing technologies that can rapidly modify genomes in a precise way and will directly enrich the nutritional status of elite varieties could hold a bright future to address the challenge of malnutrition.

## Introduction

Over two billion of the world's population is at the risk of micronutrient deficiency which resulted due to an inadequate supply of micronutrients in daily diet (1). The principal reason behind this nutrient stress is our food crops which are inadequate to supply essential nutrients as they are grown on the soils which are deficient in minerals. The agricultural production scenario in most developing countries does not address the issues related to malnutrition; rather, it focuses on increasing grain yield and crop productivity. Malnutrition is also a vital threat to the population's health and productivity and is reviewed as the largest adverse health impact of climate change. In the last few decades, the climate had altered a lot since the temperature is rising in many parts of the world, coupled with low or unexpectedly high rainfall. According to the World Health Organization and the Fifth Assessment Report of the Intergovernmental Panel on Climate Change, malnutrition is considered as one of the five largest adverse health impacts of climate change (2).

The situation of continuous degrading natural resources, farm resource constraints, and agriculture affected by changing climate in the developing countries is making a rapid rise in micronutrient deficiency in food grains, thereby increasing micronutrient malnutrition among the population, but thanks to the innovative breeding efforts by plant breeders, the agriculture in developing countries is shifting toward producing nutrient-rich food crops. This will eliminate micronutrient malnutrition in these countries, where daily diets are dominated by micronutrient-poor staple food crops. Biofortification is a strategy that can overcome nutrient deficiency more sustainably. It is a one-time investment and offers a cost-effective, long-term, and sustainable approach in fighting hidden hunger because, once the biofortified crops are developed, there are no additional costs of buying the fortificants and adding them to the food supply during processing (3). Biofortification is a process of enriching the content of vitamins and minerals in crops through genetic and agronomic interventions. Biofortification not only makes our food nutrient-rich but also reduces the cost of external amendments required to make food rich in nutrients. Approaches such as conventional plant breeding, molecular breeding, transgenic techniques, or agronomical practices provide a new avenue for the development of nutrient-rich crops. Biofortification is mainly focused on important diet-dominant foods such as cereal crops, *viz*., rice, wheat, maize, sorghum, millet, and legumes, and starchy food, *viz*., potato and sweet potatoes.

We, humans, require around 40 known nutrients in optimum amounts to live a healthy life. These nutrients are classified as macro nutrients, micronutrients, and minerals. The micronutrients, *viz*., iron, zinc, copper, manganese, iodine, selenium, molybdenum, cobalt, nickel, and vitamin A, are extremely important for a wide range of metabolic processes which ultimately lead to normal growth and development. The mineral elements such as sodium, magnesium, calcium, potassium, phosphorous, chlorine, and sulfur are classified as essential nutrients that are required in trace amounts in the body. Overall, these nutrients play a pivotal role in our physical and mental development (4). Apart from micronutrients and minerals, protein and oil content are the chief nutritional factors in food grains. Therefore, it is foremost essence to incorporate essential micronutrients, minerals, and proteins into the diets of resource-poor populations whose diet is based on cereals such as rice, wheat, cassava, and maize which contain insufficient amounts of several nutrients.

The adverse effect of climate change on nutritional food security will be primarily seen in the developing countries of Africa and South Asia. Climate change will affect not only the number of food crops but also nutritional quality. Overall, hundreds of millions of people are expected to be placed at risk of zinc, iron, and/or protein deficiencies as a result of rising CO_2_ concentrations. Recent studies proved that elevated CO_2_ concentration in the atmosphere reduces the concentrations of iron, zinc, and proteins in staple cereals, *viz*., rice, wheat, barley, and pulses. Nowadays, CO_2_ concentrations of 550 ppm, which is higher than normal, can lead to 3–11% decreases of zinc and iron concentrations in cereal grains and legumes (5). Further increase in CO_2_ concentration by 690 ppm will lead to 5–10% reductions in the concentration of iron, zinc, potassium, calcium, phosphorus, sulfur, magnesium, copper, and manganese in the majority of crops (6). The decline in zinc content due to the continuously increasing level of CO_2_ is expected to place 150–200 million people at risk of zinc deficiency (7). Mitigating the adverse effects of climate change on the nutritional quality of food crops can be achieved through breeding biofortified food crops.

Breeding biofortified crops require the identification of genetic resources with high micronutrient and mineral content from available germplasm. Most of the crops' wild relatives, landraces, and local cultivars are a rich source of these nutrients, which provide their effective utilization in the breeding program. The HarvestPlus biofortification program was started by International Food Policy Research Institute and International Center for Tropical Agriculture in collaboration with the Consultative Group on International Agricultural Research (CGIAR) centers which focused on making crops rich in vitamin A, iron, and zinc. The target crops were beans and pearl millet for iron, maize, cassava, and sweet potato for vitamin A, and wheat, rice, and maize for zinc content. The sole aim of this project is to develop micronutrient-rich crops through breeding and delivery of these nutrient-rich crops in the developing world which are mainly affected by micronutrient malnutrition. The quality traits are polygenic and are quantitatively controlled; therefore, it is difficult to improve these traits by conventional breeding. The rapid development of advanced genomic tools like molecular markers provides an effective means for improving the efficiency of plant breeding in transferring these quantitatively inherited traits. The adoption of molecular markers can remarkably facilitate the breeding programs by identifying the exact location of the genomic region/QTL controlling the trait for nutrient content. The identified QTLs can then be easily transferred to elite breeding materials if these lines have a low level of nutrient content. The usefulness of these markers based on QTL mapping was not that significant because these are based on bi-parental mapping populations. However, genome-wide association mapping studies (GWAS) offer unique opportunities to use diverse germplasm which had accumulated a large number of meiotic events as opposed to only one or a few meiotic recombinations in bi-parental mapping populations. The ability to resolve marker/trait associations depends upon the extent of linkage disequilibrium present in the panel. The markers identified to be linked with QTL have the potential to be used across breeding material for identification and introgression. In this review, we aimed to compile information on QTLs pertaining to nutrient content in important food crops, which once biofortified, may ensure good nutrition and eliminate hunger among populations, especially food-insecure children who are majorly impacted by climate-related devastations.

### QTLs for Pro-vitamin A

Vitamin A deficiency is one of the serious health problems in developing countries, leading to irreversible blindness. This situation encouraged the researchers to attempt the biofortification of plant-derived foods including pro-vitamin A carotenoids (mainly β-carotene), which led to “golden” crops. The conversion rates of β-carotene into vitamin A are reported to be high in golden rice, maize, and cassava, demonstrating that these staple crops may incorporate a higher level of nutritional impact (8, 9). Some QTLs such as *crtRB13*′*TE, crtRB1-5*′*TE-2, crtRB1-3*′*TE-1, crtRB1-5*′*TE-2, crtRB1-3*′*TE-1, LCYE, PSY1*, and *crtRb1* for pro-vitamin A have been identified on chromosome 10 in maize (10–13). In addition to this, QTL for β-carotene content has been reported in recombinant inbred line (RIL) populations through SSR markers in maize (14) (Table 1).

**Table 1:** List of important QTLs pertaining to nutritional quality in major food crops.

**Crop**	**QTL /Loci**	**MP**	**Cross(s)**	**Markers**	**Chromosome**	**Nutritional parameters in the grain**	**References**
Wheat	*QGpc-1B-2* *QGpc-4B-1.4*	RILs	Tainong 18/Linmai 6	D-1190331S-3222160 (SNP) D-1380792D-1094306(SNP)	1B 4B	Grain protein content	(15)
	*QGZn.co-5A* *QGZn.co-7A* *QGFe.co-3B.1* *QGFe.co-5A.2*	RILs	Roelfs F2007/Hong Hua Mai/./Blouk #1	1244217-1272027|F|0 (DArT) 5356706-5325178|F|0 (DArT) 1089107-1127875|F|0 (DArT) 1102433-988523 (DArT)	5A 7A 3B 5A	Zn Fe	(16)
	*QGpc.uhw-4B* *QGpc.uhw-5A.1* *QGpc.uhw-6B* *QGpc.uhw-7B.2*	RILs	Svevo/Y12-3	TG0010b(SNP) RAC875_rep_c106118-339(SNP) T.durum contig 9860-281(SNP) Kukri_c14766-484(SNP)	4B 5A 6B 7B	Grain protein content	(17)
	*qFes1* *qfes2* *qZns1* *qZns2*	Inbreds	Synthetic hexaploid wheat	Xwmc399 Xgwm157 Xwmc357 Xcfd63	4D 2D 5D 1D	Fe Zn	(18)
	*QZn 2A* *QZn7B*	Inbreds	HPAM panel genotypes	RAC875_c34757_180 (SNP) wsnp_Ex_c5268_9320618 (SNP)	2A 7B	Zn Zn	(19)
	*QGzn.ada_1B* *QGzn.sar_1B* *QGFe.ada_2B*	RILs	Adana/70711 Saricanak 98/MM S4 Adana/70711	rpt-6561 (DArT) wPt- 6434-wPt1403 (DArT) wPt-9812 (DArT)	1B 1B 2B	Zn Zn Fe	(20)
	*QZn.Across_4BS* *QFe.Across_7DS*	RILs	Seri M 82/SHW Cw176364	TP91631-TP81797 (SNP) TP43715-TP37547 (SNP)	4BS 7DS	Zn Fe	(21)
	*QZn.bhu_2B, QFe.bhu_2B* *QGPC.bhu_1A*	DHs	Berkut/Krichaff	gwm120-wPt2430 (SSR-DArT) wPt 9592–GBM 1153 (DArT-SSR)	2B 1A	Zn and Fe Protein content	(22)
	*QZn.bhu_2B* *QZn.bhu_6A* *QFe.bhu_3B*	RILs	PI 348449 (*T. spelta*)/HUW 234	989092F0 (SNP) 998265F0 (DArT) 3022954F0 (SNP)	2B 6A 3B	Zn Zn Fe	(23)
	*QGzncpk.cimmyt_2BL*	RILs	PBW 343/Kenya Swara	wPt-6174 (DArT)	2BL	Zn	(24)
Barley	*QTL.Zn*	RILs	Clipper/Sahara	Xbcd175-Xpsr108 Vrs1-XksuF15	2HS 2HL	Zn	(25)
Rice	*QTL.Fe.9* *QTL.Zn.4* *QTL.pro.1*	Inbreds	Landraces	RM215 (SSR) RM551 (SSR) RM5 (SSR)	9 4 1	Fe Zn Protein content	(26)
	*qFe3.3* *qFe7.3* *qZn2.2* *qZn8.3*	Inbreds	Germplasm lines	RM7 (SSR) RM1132 (SSR) RM300 (SSR) RM80 (SSR)	3 7 2 8	Fe Zn	(27)
	*qAAC6.1* *qAAC7.1* *qPC1.2*	RILs	Milyang23/Tong88-7	3515361-3658340(SNP) 4856196-5206110(SNP) 39162572-39234399 (SNP)	6 7 1	Amino acid content Protein content	(28)
	*qFe1.1* *qFe1.2, qZn1.1* *qFe6.1, qZn6.1* *qFe6.2, qZn6.2*	BILs	RP-Bio226/Sampada	RM562-RM11943 (SSR) RM294A-RM12276 (SSR) RM8226-RM400 (SSR) RM400-RM162 (SSR)	1 1 6 6	Fe Fe and Zn Fe and Zn Fe and Zn	(29)
	*qAA1* *qAA7*	RILs	Zhenshan 97/Delong 208	RM493–RM562 MRG186–MRG4499	1 7	Amino acid content	(30)
	*qPC6.2*	RILs	Yukihikari/Joiku 462	Indel 207-Indel 208	6	Protein content	(31)
	*qB4.1* *qCo7.1* *qCu8.1* *qK6.1* *qMn3.1* *qMg1.1* *qZn11.1* *qP11.2*	DH	R64/IR69428 (Pop1) and BR29/IR75862 (Pop2)	4333768-4349423(SNP) 7872824-7892971(SNP) 89266819-8940497(SNP) 5883472-5895767(SNP) 3005168-2732340(SNP) 115078-117345(SNP) 10907196-11001107(SNP) 11391672-11407547(SNP)	4 7 8 6 3 1 11 11	Boron Cobalt Copper Potassium Manganese Magnesium Zinc Phosphorous	(32)
	*qGPC1.1* *qSGPC2.1* *qSGPC7.1*	BILs	ARC10075/Naveen	93237905-93229368(SNP) 93256429-93260438(SNP) 93225742-93256949(SNP)	1 2 7	Grain protein content	(33)
	*qFe7* *qZn7*	DH	Goami2/Hwaseonchal	RM10-RM1973 (SSR) RM10-RM1973 (SSR)	7	Fe Zn	(34)
	*qFe1.2* *qFe11.1* *qZn2.1* *qZn3.2* *qFe3.2* *qFe4.1* *qZn5.1* *qZn12.1*	BILs	Swarna/*O. nivara* (IRGC81832) P1 Swarna/*O. nivara* (IRGC81848) P2	RM243-RM81A (SSR) RM332-RM287 (SSR) RM250-RM535 (SSR) RM55-RM520 (SSR) RM520-RM514 (SSR) RM241-RM348 (SSR) RM153-RM413 (SSR) RM415-RM19 (SSR)	1 11 2 3 3 4 5 12	Fe Fe Zn Zn Fe Fe Zn Zn	(35)
	*qFe10.1* *qZn6.2* *qZn7.1*	MAGIC	MAGIC Population	S10_13883426 S6_2939487 S6_29350591	10 6 7	Fe Zn Zn	(36)
	*qFe 6* and *qZn 8*	BILs	IR 7586/Ce 258 IR 7586/Zhongguangxiang1	RM3- RM340 (SSR) RM407- RM152 (SSR)	6 8	Fe Zn	(37)
	*qFe 1.2 (gene OsYSL1)* *qFe 5.1 (gene OsZIP6)* *qFe 7.2 (gene OsZIP8)*	RILs	Madhukar/Swarna	RM490 (SSR) RM574 (SSR) RM8007 (SSR)	1 5 7	Fe	(38)
	*qPro-2* *qPro-10* *qLip-6*	DHs	Cheongcheong/Nagdong	RM5619-RM1211 (SSR) RM24934-RM25128 (SSR) RM586-RM1163 (SSR)	2 10 6	Protein content C Protein content LC	(39)
	*qPro-2*	DHs	Cheongcheong/Nagdong	RM12532-RM555	2	PC	(40)
	*qFe 2, qZn 5, qCo 1, qCu 2, qMn 7*, and *qMo 7*	BILs	Teqing/Lemont	RM452, RM421, RM490, RM6378, RM214, and RM11 (SSR)	2, 5, 1, 2, 7and 7	Fe, Zn, Co, Cu, Mn, Mo	(41)
	*OsZIP8a* *OsNAC* *OsZIP4b*	RILs	IRRI 38/Jeerigesanna	RM8007 (SSR) - -	7 3 8	Zn	(42)
	*qPC 1*	RILs	Zhenshan97/Nanyangzhan	RM472- RM104	1	Protein content	(43)
Maize	*crtRB13'TE*	Inbred	1.V335/HP465-30 2.V345/HP465-35	crtRB1-3'TE-1 (SSR)	10	Pro-vitamin A	(12)
	*crtRB1-5'TE-2* *crtRB1-3'TE-1*	Inbreds	Hp321-1	crtRB1-5'TE-2 (SSR) crtRB1-3'TE-1 (SSR)	10	Pro-vitamin A	(13)
	*LcyE* *crtRB1-3'TE*	F_2_ F_3:4_	L1-L5 H1-H15	LcyE-5'TE (SSR) LcyE-3'TE (SSR) crtRB1-3'TE (SSR)	10	Pro-vitamin A	(10)
	*LCYE* *PSY1* *crtRb1*	Inbreds	130 Tropical Inbred lines	LCYE-5'TE (285 Indel) LCYE-SNP (G-C SNP) LCYE-3'TE (8 bp Indel) PSY-SNP7 (A-C SNP) PSY1-1D1 (378 Indel) crtRB1-5'TE (397/206 bp Indel) crtRB1-InDel4 (12 bp Indel) crtRB1-3'TE (325/1250 bp Indel)		Pro-vitamin A	(11)
	*crtRB1*	RILs	1.B73/BY804 2.A619/SC55 3.K13/B77 4.K13/SC55	umc1506-bning1028 umc1506-crtRB1 crtRB1 crtRB1	10	β-carotene, βC/βCX, βC/Z and βC/ALL	(14)
	*sQTL4.2* *sQTL4.1* *sQTl3.1*	RILs	Ye478/Wu312	umc1620-umc1994 (SSR) umc1346-bnlg2291 (SSR) mmc0132-umc1504 (SSR)	4 4 3	Zn Mn Mg	(44)
	*qZn5,qMn1, qCo3, qCu8, qK4, qMo1, qNa5, qP4, qS1*	RILs	B73/IL14H	PZA02411.3, PZA02135.2, PZA01925.1, PZA03698.1, PZA01751.1, PZA02269.3.4, PZA01327.1, PZA01751.2, PZA02698.3 (All SNP)	5, 1, 3, 5, 8, 4, 1, 5, 4, 1	Zn, Mn, Co, Cu, K, Mo, Na, P, S	(45)
Pearl millet	*qFe1/54 and qZn1/54*	RIL	ICMS 8511-S1-17-2-1-1-B-P03 _ AIMP 92901-S1-183-2-2-B-08	Pgpb10531-pgpb9130 (DArT)	LG1	Fe and Zn	(46)
	*Fe and Zn*	Inbreds	130 germplasm lines	Xpsmp2261, Xipes0180, Xipes0096		Fe and Zn	(47)
Common Bean	*MQTL_Fe,Zn 1.1* *MQTL_Fe,Zn 6.1* *MQTL_Fe,Zn 6.1*	RILs	7 Populations		1 6 6	*Fe and Zn*	(48)
	*Seed coat Fe* *Seed coat Zn*	BILs	Cerinza/G10022	Pv-at03, ATA16, ATA26 ATA247, ATA10	4, 2, 3 8, 6	Seed coat Fe Seed coat Zn	(49)
	*Ca1, Ca7, Ca9* *Mg7* *Pt5, Pt7* *DF6, DF7*	RILs	Xana/Cornell 49242	McatEtc, P gene, McagEac P gene OD12^800^, SAS8 DBD (AC), P gene	1, 7, 9 7 5, 7 6, 7	Ca Mg Protein content Dietary fiber	(50)
	*Fe_cont8.1* *Zn_cont2.1, 5.1, 5.2 and 7.1*	BILs	Cerinza/G10022	BMc316 (SSR) PV109, BM155, BMd28 and PV35 (SSR)	8 3, 5, 5, 7	Fe Zn	(51)
	*Fe-AAS2a* *Fe-AAS6c* *Zn-AAS2c* *Zn-AAS7c*	RILs	G21242/G21078	E0403A (SSR) N0401B (SSR) Pv11 (SSR) BM239 (SSR)	2 6 2 7	Fe concentration Zn concentration	(52)
	*MnQTL1.1* *MnQTL3.2* *MnQTL3.3*	RILs	CDC Redberry/ILL7502	SNP	LG LG3 LG3	Mn content	(53)
Lentil	*FeQTL1.2, 2.2, 4.2, 5.2, 6.2, and 7.1*	RILs	ILL8006/CDC Milestone	15SNP, 81 SNP, 40 SNP, 239 SNP, 4 SNP and 12 SNP	1, 2, 4, 5, 6, 7	Fe concentration	(54)
	*Zn content*	Inbreds	143 diverse germplasm lines	LcC06739p564(SNP) LcC04105p1090 (SNP)	3 2	Zn content	(55)
	*SeQTL2.1, 5.1, 5.2, 5.3*,	RILs	PI320937/Eston	SNP	2,5,5,5	Selenium content	(56)
	*qFe uptake*	RILs	ILL8006-BM/CDC Milestone	–	–	Fe uptake	(57)
	*121 QTLs for Mn and Zn concentartion*	RILs	CDC-Redberry/ILL7602	–	–	*Mn and Zn concentartion*	(58)
Pea	*[B]-Ps5.1, [Ca]-Ps5.1, [Mg]-Ps5.1, [S]-Ps5.1* *[Fe]-Ps7.1, [Zn]-Ps7.1* *[K]-Ps5.1* *[Mn]-Ps5.1* *[Mo]-Ps5.1* *[P]-Ps3.1*	RILs	Aragorn/Kiflica	TP61763 (SSR) TP44143 (SSR) TP55189 (SSR) tip_SNP2_V (SNP) TP42330 (SSR) TP75231 (SSR)	5 7 5 5 5 3	B, Ca, Mg SFe, ZnKMnMoP	(59)
	*QTL.Fe1* *QTL.Fe2* *QTL.Fe3*	RILs	Carrera/CDC Striker	Sc1203_101100 and PsC17710p220 Sc9618_162688 and PsC4833p179 Sc2559_48386 and PsC908p622 (All SNP)	3 4 7	Fe concentration	(60)
	*QTL FEBIO*	RILs	1-2347-144/CDC Meadow	PA-P (SNP)	5	Fe bioavailability	(61)
Chickpea	*CaqFe1.1* *CaqZn2.1* *CaqFZ4.1* *CaqFZ5.1* *CaqFZ7.1*	Inbreds	92 Germplasm lines	SNP53-SNP55 SNP110 SNP300 SNP413 SNP471-SNP472	1 2 4 5 7	Fe Zn Fe and Zn Fe and Zn Fe and Zn	(62)
	*QTL.PC*	Inbreds	187 Germplasm lines	TR26.205 (SNP), CaM1068.195 (SNP)	3, 5	Protein content	(63)
	*QTL.Zn* *QTL.Fe*	Inbreds	94 Germplasm lines	Cav1Sc25.1p2052607 Cav1Sc19.1p1556596	4 1	Zn Fe	(64)
Peanut	*qOCB3* *qPAA8* *qOAA3* *qEAA5* *qBAB9* *qAAB9* *ASAA4*	F_2_	Zhonghua10/ICG12625	AHGS1788-pPGSseq14C11 PM54-pPGPSseq2G3 TC4E10-ARS744 GM1577-TC6E1 AHGS1969 AHGS1969 GM2480	B3 A8 A3 A5 B9 B9 A4	Oil content Palmatic acid Oleic acid Eicosenic acid Behenic acid Arachidic acid Stearic acid	(65)
	*S_mqPAb09_4*, *S_mqAA_b098* *S_mqGA_b09_1* *S_mqSAa07_1* *S_mqBA_a09* *S_meLA_b08_2*	RILs	Sunoleic97R/NC94022 (Population S)	FAD2B RN34A10 FAD2A Seq2A06	B9 A7 A9 B8	Palmatic acid, Arachidic acid, Gadoleic acid Stearic acid Behenic acid Lignoceric acid	(66)
	*T_mqSA_b04_1*, *T_mqAA)b04_1*, *T_mqLA_b04_1* *T_mqGA_b04_1, T_mqBA_b04_4* *T_mqPA_a09_4*	RILs	Tifrunner/GTC20 (Population T)	PM15 TC4H07 FAD2A	B9 B4 A9	Stearic acid Arachidic acid, Lignoceric acid Gadoleic acid, Behenic acid Palmatic acid	(66)
	*mqQA181*, *mqLA182, mqOLR* *mqOC*	RILs	Sunoleic97R/NC94022 (S population)	ahFAD2B GM1878	B9 A5	Oleic acid, Linoleic acid, Oleic/Linoleic acid Oil content	(67)
	*mqQA181*, *mqLA182, mqOLR* *mqOC*	RILs	Tifrunner/GT-C20 (T population)	ahFAD2A GM2690-1	B9 B8	Oleic acid, Linoleic acid, Oleic/Linoleic acid Oil content	(67)
Mung bean	*qFe4.1, qZn6.4*,	RILs	ML776/Sattya	PVBR82 (SSR), CEDG248 (SSR)	4,6	Fe, Zn	(68)
	*SDPAP4.1* *SDIP10.1* *SDTP4.1*	F_2_	V1725BG/Aus TRCF 321925	CEDG139-MB-SSR179 (SSR) VR226-CEDG097 (SSR) Bmd25-MB-SSR179 (SSR)	4 10 4	Low phytic acid Inorganic phosphate Total phosphorus	(69)
Soybean	*qCys-7-2* *qMet-8-1* *qSAA-15-1* *qPC-8-1*	RILs	Kefeng no. 1 and Nannong 1138-2	Bin 148 (SNP) Bin 34 (SNP) Bin 124 (SNP) Bin 37 (SNP)	7 8 15 8	Cysteine Methionine Sulfur containing amino acids Protein content	(70)
	qPRO001 qOIL001 qPAL002 qOLE003 qLIN001 qGLU001 qALA001 qCYS001 qVAL001 qHIS001	RILs	Hamilton/Spencer	ss249909538–ss249919445 ss246100375–ss245879277 ss245914593–ss245908292 ss249909538–ss249506152 ss245914593–ss245790648 ss245914593–ss245908292 ss246100375–ss245879277 ss245898080–ss245908292 ss249909538–ss249919445 ss245898080–ss245908292	18/G 6/C2 6/C2 18/G 6/C2 6/C2 6/C2 6/C2 18/G 6/C2	Protein Oil Palmitic Oleic Linolenic Glutamic Alanine Cysteine Valine Histidine	(71)
	*qIF5-1*	RILs	Huachun 2/Wayro	Bin 799-800 (SNP)	5	Isoflavone content	(72)
	*qProt_Gm20* and *qLsy_Gm20* *qThr_Gm20, qMet_Gm20 and qMet+Cys_Gm20* *qCys_Gm10*	RILs	Benning/Danbaekkong	GSM0012 (SNP)-satt354 (SSR) GSM0012-BARC-020713 (SNP) satt592 (SSR)-BARC043247 (SNP)	20 20 10	Protein content and Lysine content Threonine, Methionine and Methionine + Cysteine content Cysteine content	(73)
	*suc1, suc3, suc2*	F_3_	MFS-553/PI243545	ss245668753, ss249186914, ss246796276 (SNP)	5, 9, 16	Sucrose content	(74)
	*qPC*	RILs	1.R05-1415/R05-638 2.V97-1346/R05-4256	satt451-satt614 (SSR) ss250447161-ss250327854 (SNP)	20 20	Protein content	(75)
	*qOC*	RILs	R05-1415/R05-638	satt451-satt614 (SSR)	20	Oil content	(76)
	qCa-8-1 qMg-13 qZn-11-1 qFe-3 qP-11	RILs	Kefeng1/Nannong1138-2	sat_162-AW132402 satt335-satt522 satt251 satt675-satt237 satt197	8 13 11 3 11	Calcium content Magnesium Zn Fe Phosphorous	(77)
	*qPRO_B1* *qOIL_C1,J,O*	RILs	SD_02_-4-59/A_02_-381100	BARCSoYSSR-17-0621 (SSR) Sat_140, Sat_350, satt581 (SSR)	17 4,16,10	Protein content Oil content	(78)
	*qPRO_13_1*, *qPRO_13_1*	F_2:3_	1. Jidou 12/ZYD 2738 (*G. soja*) 2. Jidou 9/ZYD 2738 (*G. soja*)	satt114 (SSR) satt114	13	Protein content	(79)
	*qCys* and *qCys-Met* *qMet*	RILs	1.Williams 82 /DSR-173 2. Williams 82/NKS19-90 3. Williams 82/Vinton81	BARC-038869-07364 - BARC-039753-07565 (SNP) BARC-018461-02916 – BARC-066103-17539 (SNP)	20	Cysteine and Cystein + Methionine content Methionine content	(80)
	*qIF20-2*	RILs	Luheidou2/Nanhuizao	M943408-M941848 (SLAF)	20	Isoflavone content	(81)
	*Ca5*	F_2:4_ F_2:5_	PI 408052B/PI 408052C PI408052B/KS 43035P	Sat_290- satt115	18	Calcium content	(82)
	*qPRO001* *qOIL008* *qPAL002* *qSTEL001* *qOLE003* *qLINL003* *qLINN007*	RILs	MD96-5722/Spencer	ss248293401-ss248275088 (SNP) ss248308943-ss248309108 (SNP) ss249629157-ss249621644 (SNP) ss248977568-ss248979552 (SNP) ss249037210-ss249039670 (SNP) ss248981433-ss248993887 (SNP) ss249010538-ss249039670 (SNP)	14 14 18 16 16 16 16	Protein content Oil content Palmatic acid Stearic acid Oleic acid Linoleic acid Linolenic acid	(83)
	*qPRO7-5* *qPRO20-1*	RILs	Charleston/Dongnong 594	satt358-Sat_001 (SSR) satt331-satt173 (SSR)	7 20	Protein content	(84)
*Brassica napus*	*qoil.1* *qpro-1* *qoil+pro-1*	DH	SGDH14/cv. Express	pP12638473-p12699181 (SNP) p18005556-p11609327 (SNP) p12699181-scaff_17119_1_p115218	A08 A07 A08	Oil content Protein content Oil and Protein content	(85)
	*BnaA05g 23520* *BnaA05g 23930*	DHs	RIL324/RIL622	UQSNP0001565 UQSNP0001759		Oleic and Linoleic acid	(86)
	*cqOC-A8-2* *uqFA-C3-3* *uqFA-A8-4*	DH	Tapidor × Ningyou 7		A8 C3 A8	Oil content C16:0/C18:0/C18:1/ C18:2/C20:0/C20:1/ C22:0/C22:1/C16:0/ C18:0/C18:1/C18:2/ C18:3/C20:0/C20:1/ C22:0/C22:1/	(87)
	*Gene BrFAD5* *Gene BrFAD7*	DHs	YS 143 /Nai Bai Cai	Bra027203 Bra034863	A05 A05	Erucic acid Oleic and Linoleic acid	(88)
	qOIL-A10a qC16:0-C8b qC18:0-C3e, qC18:1-C3c, qC18:2-C3c, qC18:3-C3c, qC20:0-C3d	DHs	Polo 9/Topas	BnGMS288-311/CB10536-163 em1/bg9-434/bg23/pm59-285 pm88/pm45-177/odd3/pm3-399	A10 C8 C3	Oil content Palmatic acid Stearic, Oleic, Linoleic, Linolenic, Arachidic acid	(89)
	*Oil content* *Protein content* *Erucic acid* and *Stearic acid* *Linoleic acid* *Glucosinolate*	DHs	Tapidor/Ningyou7	Bn-scaff_23761_1-p249628 (SNP) Bn-scaff_17119_1-p349622 (SNP) Bn-scaff_15794_1-p347392 (SNP) Bn-A02-p10850012 (SNP) Bn-scaff_15794_1-p437864 (SNP)	C03 C03 C03 A02 C03	Oil content Protein content Erucic and Stearic acid Linoleic acid Glucosinolate	(90)
	*OilC3-3*	DHs	Tapidor/Ningyou7	Bn-scaff_23761_1-p249628 (SNP)	C03	Oil content	(91)
	*PRT.C6.w.1* SUL.A2.w.1 OLA.A9.w.1 OIL.C3.w.1 LIA.C3.w.1 GSL.A2.w.1 ERA.C3.w.1	Inbreds	405 Inbred lines	Bn-ctg7180014756759-p1575 Bn-ctg7180014748062-p8451 Bn-Scaffold000110-p349432 Bn-ctg7180014717095-p1564 Bn-ctg7180014726380-p989 Bn-ctg7180014748062-p8451 Bn-ctg7180014717095-p1564 (SNP markers)	C6 A2 A9 C3 C3 A2 C3	Protein content Sulphur content Oleic acid Oil content Linolenic acid Glucosinolate Erucic acid	(92)
	qLysC-16-3 qThrC-12-5 *qMetC-9-5*	BC	BC_1_F_1_ (DHs/Tapidor) BC_2_F_1_ (DHs/Ningyou7)	HBR057/HBR047 EM18ME6-220/NA12C03HBR096/IGF5385F	C6 C2 A9	Lysine content Threonine content Methionine content	(93)
	*qOC-2* *qPC-1* *qOAC-2* *qGLC-1*	RILs	827R/Darmor_Sin	CB10369-220 - me5em16-170 me1em1-400 - me4em7-400 Ol09-A06-400 - me2em10-240 me7em11-470 me8em20-230 (SRAP markers)	11 1 12 10	Oil content Protein content Oleic acid Glucosinolate	(94)
	*qA8-5* *qC3-3*	DHs	Tapidor/Ningyou7	IGF1108c-sR7178 IGF0235b-BRMS-093	A8 C3	C16:0/C18:0/C18:1/ C18:2/C18:3/C20:0/ C20:1/C22:0/C22:1	(95)
	*qC16:0, qC18:1, qC18:2, qC18:3, qC20:1*	DHs	Tapidor/Ningyou7	HBr015 (A8) and JICB0633 (C3)	A8 and C3	C16:0, 18:1, C18:2, C18:3, C20:1	(96)
	*FAD3A* and*FAD3C* genes	Inbreds	21 Cultivars of winter and spring rape	LinAR-LinAF and LinCr-LinCF (dCAPS markers)		Low Linolenic acid	(97)
	BnFAD2-C5	Inbred	Xiangyou	YG-C5-FAD2-F/YG-C5-FAD2-R	C5	High oleic acid	(98)
	*qOil_N19* *qC160180_N9* *qC181_N9* *qC182_N9* *qC183_N6* *qProtein_N16*	DHs	DH12075 / YN01-429	- - - - - -	N19 N9 N9 N9 N6 N16	Oil content C16:018:0 C18:1 C18:2 C18:3 Protein content	(99)
	*qArgC-8-5* *qHisC-4-3* *qGluC-1-1* *qGlyC-4-1* *qProC-1-1* *qAlaC-7-3* *qAspC-9-4*	BC	BC1F1 1 (DHs/Tapidor) BC1F1 2 (DHs/‘Ningyou7)	*HAU348/B034P14-1-1* HS-k02-2/HBr094 znS13M26-100/CB10081 HBr094/CNU256 znS13M26-100/CB10081 znS13M26-340/JICB0571 HBr075/JICB0516	*A8* A4 A1 A4 A1 A7 A9	Arginine content Histidine content Glutamic acid Glysine content Protein content Alanine content Aspartic acid	(100)
	*BnaFAD2 gene*	Inbred	Tapidor	BnaC.FAD2.a BnaA.FAD2.b	A5 A1	High Oleic and low PUFA	(101)
*Brassica carinata*	*qPRO10* *qEru.C4-1* *qLEN.C4-1* *qOLE.C4-1* *qLEI.B8-4*	DHs	Y-BcDH64/W-BcDH76	100059607 (DArTseq) 100036778 (DArTseq) 100065508 (DArTseq) 100026342 (DArTseq) 100035893 (DArTseq)	B7 C4 C4 C4 B8	Protein content Erucic acid Linolenic acid Oleic acid Linoleic acid	(102)
	PRO-WH13 OLE-WH14 OC-WH13 LEN-WH14 ERU-WH13	Inbreds	81 diverse accessions	5121285 (DArTseq) 5859309S (DArTseq) 5121285 (DArTseq) 5863483-1S (DArTseq) 5121285 (DArTseq)	C8 B2 C8 B3 C8	Protein content Oleic acid Oil content Linolenic acid Erucic acid	(102)
*Brassica juncea*	TGLC-S GNA-S SIN-S IBE S NEO-S NAS-S TOC OIL	RILs	NUDH-YJ-04/RL-1359.	SB3739a -SB3739b (SSR) NA14B05- NIA138 (SSR) CNU566- NA12D08 (SSR) NIA010 -NIA046 (SSR) NIA045 -NIA043 (SSR) NA12H09- NA12G08 (SSR) CNU111 -CNU483 (SSR) NIA044- CNU402b (SSR)	J18 J1 J4 J9 J7 J4 J6 J9	Glucosinolate Gluconapin Sinigrin Glucoiberin Gluconeobrasscin Gluconasturtin Tocopherol Oil content	(103)
	*FAE1.1* *FAE1.2*	Inbreds andBC	18 Inbred lines PM24/Pusa Vijay PM30/Pusa Bold	CAPS591, CAPS1265 CAPS237	A8 B7	Low erucic acid	(104)
*Brassica rapa*	*BnFAE1 gene*	BC	Tori-7 / Kirariboshi	BnFAE1.1-dcapsF BnFAE1.1-3UTR-cR	A C	Low erucic acid	(105)
	*Gene BrFAD5* *Gene BrFAD7*	DHs	YS 143 /Nai Bai Cai	Bra027203 Bra034863	A05 A05	Erucic acidOleic and linoleic acid	(88)
*Brassica oleracea*	BoFAD3-2 gene		alboglabra	BoFAD3-2FY1 - BoFAD3-2FY2 (gene specific markers)		Low α-linolenic acid	(106)

### QTLs for Fe and Zn Content

Iron (Fe) and zinc (Zn) are among the essential micronutrients that are often lacking in human diet (4). According to WHO, over 30% of the population is anemic. These micronutrients play a wide range of metabolic functions required for normal growth and brain development. Zinc is an essential nutrient in the proper functioning of the immune system. Infants, small children, and pregnant and lactating women are at a higher risk of Fe and Zn depletion. Therefore, it is important to raise the level of Fe and Zn in the daily diet of children and women to save them from malnutrition. Cereals biofortified with Fe and Zn can eliminate the problem of malnutrition. These traits (Zn and Fe) are polygenic in nature, and genetic variation for these traits exists in landraces and progenitor species of important food crops. Breeding strategies are therefore focusing on novel approaches to broaden the genetic base using wild/related species and landraces and identifying genetic control and their effects (107). Several genetic mapping populations have been developed in the past few years to dissect Fe- and Zn-related traits. In wheat, various studies have reported QTL for high grain Fe and Zn concentrations on chromosomes 1A, 1D, 1B, 2A, 2B, 3B, 3D, 4B, 5A, 6A, 6B, 7A, 7B, and 7D in hexaploid wheat (16, 18, 20–24) and on chromosome 2 in barley (25) (Table 1). Recently, Velu et al. (19) evaluated the HarvestPlus Association Mapping panel across a range of environments in India and Mexico. GWAS analysis revealed two larger QTL regions on chromosomes 2 and 7 for large grain Zn content.

In rice, QTLs (*qFe 1.2, qFe 2*, and *qFe 6*) were identified on chromosomes 1, 2, and 6 for Fe content (37, 38, 41) and on chromosomes 5, 8, and 7 (*qZn5, qZn 8*) for Zn content (37, 41, 42). Most of the QTLs for Fe and Zn content were identified through single-nucleotide polymorphism (SNP) and DArT-SSR markers in RIL and BIL populations, but some of them were also from DH lines (Table 1). Recently, many new QTLs for Fe and Zn content have been mapped mainly through SSR markers in DH and BIL mapping populations (26, 27, 29, 34–36) (Table 1). Maize hybrids and varieties having a high yield potential along with 25–30% more Fe and Zn than common cultivars have been developed as part of the HarvestPlus program (108). Pearl millet is an important nutri-cereal rich in Fe and Zn. Recently, Kumar et al. (46) and Anuradha et al. (47) have identified QTLs for grain Fe and Zn content in RILs and 130 pearl millet germplasm lines (Table 1).

Pulses have a uniformly higher amount of Fe and Zn compared to the cereals and are a better source of micronutrients. Izquierdo et al. (48), using a meta-QTL analysis in 7 populations, identified three major QTL regions governing seed Fe and Zn content and concentration in seeds. Many other QTLs for Fe and Zn content (Table 1) have also been reported in common bean using SSR markers (49, 51), in lentil using SNP markers (54, 55, 58), and in peas using SSR (60) and SNP markers (59). In chickpea, Upadhyaya et al. (62) and Diapari et al. (64) identified QTLs for Fe and Zn content using SNP markers in germplasm lines (Table 1). Similarly in mungbean, two QTLs, *viz*., *qFe4.1* and *qZn6.4* were mapped on chromosomes 4 and 6, respectively, using SSR markers in a RIL population (68). In soybean, Ning et al. (77) reported two QTLs, *qZn11.1* and *qFe3*, on chromosomes 11 and 3, respectively, for seed Zn and Fe content. In lentils, the diversity of genes relating to iron and zinc contents in seeds was studied using molecular markers, with implications for biofortification (109–111).

### QTLs for Oil Content

Oil content is a classic but significant yield trait in groundnut, peanut, soybean, and various *Brassica* species. The fatty acid composition of various edible oil crops has various parameters such as nutrition, oxidative stability, and shelf-life that correlate with overall oil quality. So, modifying the oil content of the majority of vegetable oil crops is one of the breeding objectives, while breeding vital, healthy, and desired fatty acids should be envisaged for improving oil quality in the specific genotypes. Generally, a higher content of oleic acid and a lower content of linoleic acid (high oleic/linoleic acid ratio) in cooking/edible oil is reported to be healthier for human consumption. Many QTLs (Table 1) have been reported for oil content in diploid and amphiploid species of *Brassica* such as *Brassica napus* (87, 89–91, 94, 99) and *Brassica carinata* (102). In *Brassica juncea*, QTLs for glucosinolate, gluconapin, sinigrin, glucoiberin, gluconeobrasscin, gluconasturtin, and tocopherol content were mapped in RILs on chromosomes (J18, J1, J4, J9, J7, and J6, respectively) using SSR markers (103). Researchers have developed “canola-grade” mustard varieties containing low levels of erucic acid and glucosinolates. The erucic acid content in *Brassica*s is controlled by the *FAE1* gene. CAPS markers for genes *FAE1.1* and *FAE1.2* were developed and successfully used in breeding programs in the improvement of *B. juncea* (104) and *B. rapa* (105), but when compared to simple PCR-based markers, CAPS markers are labor- and cost-intensive, which restrict their routine use by breeders. Recently, Saini et al. (112) assessed polymorphism in the upstream region of FAE1.1 and FAE1.2 genes, across the low erucic acid and high erucic acid genotypes, and developed PCR-based markers (FAE1.1P and FAE1.2P) based on this variability. These markers can be effectively used in marker-assisted selection for the development of low-erucic-acid varieties in B. juncea. In *B. Oleracea*, gene *FAD3-2* for low α-linolenic acid has been tagged by a gene-specific marker (106) (Table 1). Peanut and soybean are extensively cultivated in many parts of the world and are an important source of edible oil. Researchers are identifying QTLs for the development of peanut genotypes with good oil quality and desired fatty acid composition. Huang and co-workers (65) reported QTL *qOCb3* for oil content in the F_2_ population, whereas Pandey and co-workers (67) reported a meta-QTL (*mqOC*) for oil content in the RIL population (Table 1). In soybean, seed oil content is under polygenic control and is sensitive to environmental effects. QTLs for oil content flanked by SSR marker (76, 78) and SNP markers (83) have been reported in the RIL population.

### QTLs for Fatty Acid Content

The trait associated with oil quality, measured in terms of its fatty acid composition, is an important agronomic trait that can eventually be tracked using molecular markers. Oils provide essential fatty acids along with an array of vitamins. Researchers have identified QTLs for major saturated (palmitic acid, arachidic acid, stearic acid, behenic acid, *etc*.) and unsaturated fatty acids (oleic acid, linoleic acid, linolenic acid, erucic acid, etc.) in major oilseed crops. The QTLs for major fatty acids will be discussed below.

*Palmitic acid* (C16:0**)** is the most common saturated fatty acid found in animals, plants, and microorganisms and is also the precursor to longer fatty acids (113). Many QTLs have been reported in major oilseed crops such as peanut (65, 66), soybean (83), and *B. napus* (87, 89, 95, 96, 100) for this trait. The details of the marker linked to palmitic acid content are given in Table 1.

*Stearic acid* (C18:0) is a saturated fatty acid, and its ester is one of the most common saturated fatty acids found in nature following palmitic acid. Many QTLs controlling stearic acid content have been reported in several oil crops. In soybean, a major QTL (*qSTEL001*) has been reported using SNP markers on chromosome 16 in RIL population (83). In *B. napus*, a QTL (qC18:0-C3e) was reported on chromosomes C8 and C3 using DH lines (89). In peanuts, QTLs such as *ASAA4, S_mqSAa07_1*, and *T_mqSA_b04_1* have been reported in F_2_ and RILs on chromosomes A4, A7, and A9 (65, 66).

*Arachidic acid* (C20:0) is a saturated fatty acid present in peanut seeds. Several QTLs have been identified in soybean, peanut, and *Brassica* species pertaining to it. In *B. napus*, QTL qC18:3-C3c was present on chromosomes C3 and C8 in DH lines (87). QTLs such as *qAAB9, S_mqAA_b098*, and *T_mqAA_b04_1* in peanut were identified on chromosome B9 using F2 and RILs population (65, 66). The details of the marker linked to arachidic acid content are given in Table 1.

*Oleic acid* (C18:1) is a naturally occurring fatty acid among various animal and vegetable oils. Oleic acid accounts for about 80% of peanut oil. Norden et al. (114) have identified two high oleic acid peanut lines (F435-2-1 and F435-2-2) through a systematic breeding program in Florida. In the same year, genetics of this trait came before the world. Moore and Knauft (115) identified two homozygous recessive mutant genes FAD2A and FAD2B for high oleic acid content, but this was at the cost of lower linoleic acid. As time progressed, many reports have come out on mapping of this trait. In peanut, QTLs for high oleic acid content have been reported by many researchers (Table 1). Through the DArTseq marker, a QTL (*OLE-WH14*) was reported on chromosome B2 in inbred lines and another QTL (*qOLE.C4-1*) was also reported on chromosome C4 in DH lines in *B. carinata* (102). Similarly, in *B. Napus*, QTLs (*BnaA05g 23520, BnaA05g 23930, Gene BrFAD5*, and *Gene BrFAD7*) were detected for oleic acid using DH lines as mapping populations (Table 1). In soybean, a QTL (*qOLE003*) was reported using SNPs in the RIL population. In peanut, a QTL (*qOAA3*) was identified in F_2_ and another QTL (*mqQA181*) in RILs population on chromosomes A3 and B9 (Table 1).

*Linoleic acid* (C18:2) is about 40% in normal peanut lines. Pandey and co-workers (67) reported the relationship of FAD2 genes with peanut oil quality and suggested that FAD2B contributed higher phenotypic variance for linoleic acid than the FAD2A alleles. Recently, Hu et al. (116) detected the two main QTLs for linoleic acid content located in linkage groups A09 and B09 in peanut. They reported that SNP markers, *viz*., Marker2575339 and Marker2379598 in B09, were associated with oleic acid and linoleic acid in seven environments and Marker-4391589 and Marker-4463600 in A09 in six environments. The QTLs for linoleic acid were identified in other crops, too, *viz*., soybean (*qLINN007*), *B. carinata* (LEN-WH14, *qLEI.B8-4*), and *B. napus* (*uqFA-C3-3, uqFA-A8-4, qC182_N9, FAD7, FAD3A*, and *FAD3C* genes) using RILs, DHs, and inbred lines (Table 1).

### QTLs for Protein and Amino Acid Content

Pulses are important sources of proteins, important nutrients, and calories, particularly in diets of peoples living in developing countries. Paying less attention to the genetic enhancement of pulses is likely to have a noticeable impact on global food and nutritional security (117). Advances in sequencing technologies have made significant improvements in the breeding lines of important pulses such as pigeonpea, chickpea, and groundnut; however, the pace in improvement does not match that of cereals. Importantly, the consumption of pulses has been shown to have several positive health effects that are inherently tied to their nutritional qualities (118). The development of improved cultivars of pulses can be accelerated through the identification and the deployment of the gene(s)/QTLs of nutritional quality traits. In chickpea germplasm lines (Desi and Kabuli), two significant QTLs for protein content linked with SSR markers TR26.205 and CaM1068.195, present on LG3 and LG5, were reported (63). In soybean, major QTLs *(qProt_Gm20, qPRO_13_1, qPRO001*, and *qPC)* for protein content flanked by SNP and SSR markers were identified in RILs (66, 73, 79, 83). In *B. carinata*, QTLs for protein content were identified (*qPRO10* and PRO-WH13) through DArTseq marker using DH and inbred lines (102) (Table 1). Among cereals, rice and wheat are also sources of protein in daily diets, though in limited quantity. The major effect of QTLs for grain protein content has been mapped in rice (28, 33, 39, 43) and Wheat (15, 17, 22) (Table 1).

Protein is composed of amino acids; these amino acids are the “building blocks” of the body, and improving essential amino acids is one of the better prospects envisaged. Some QTLs corresponding to essential amino acids have been identified in important food crops. Amino acid contents such as those of lysine, threonine, methionine, and cysteine were improved in soybean through the identification of QTLs in different RIL populations (70, 71, 73, 80). In *B. napus*, QTLs were identified for lysine, threonine, and methionine content (qLysC-16-3, qThrC-12-5, and qMetC-9-5) in DH lines on chromosomes C6, C2, and A9 (93). In *B. napus*, arginine, histidine, glutamic acid, glycine, alanine, and aspartic acid content was improved through QTLs such as *qArgC-8-5, qHisC-4-3, qGluC-1-1, qGlyC-4-1, qProC-1-1, qAlaC-7-3*, and *qAspC-9-4* in BC lines on chromosomes A1, A4, A7, A8, and A9 (100). The details of the linked markers to these QTLs are given in Table 1.

### QTLs for Minor Elements

Trace elements are required in a minimal amount, and these micronutrients are vital as they are often acting as catalysts in chemical reactions. In rice, QTLs, *viz*., *qCo 1, qCu 2, qMn 7*, and *qMo 7* in BILs for Co, Cu, Mn, and Mo on chromosomes 1, 2, 5, and 7, have been identified (41). Similarly, in maize, QTLs (*qMn1, qCo3, qCu8, qK4, qMo1, qNa5, qP4*, and *qS1*) for some trace elements (Mn, Co, Cu, K, Mo, Na, P, and S) have been identified on chromosomes 1, 3, 4, 5, and 8, respectively, by Baxter et al. (45). Furthermore, Ates et al. (56) identified four QTLs for selenium content using SNP markers in lentil (Table 1). QTLs [(K)—*Ps5.1*, (Mn)—*Ps5.1*, (Mo)—*Ps5.1*, and (P)—*Ps3.1*] for minor nutrients were identified through SSR markers in pea RILs (59). Sompong et al. (33) reported QTLs for low phytic acid (*SDPAP4.1*), inorganic phosphate (*SDIP10.1*), and total phosphorus (*SDTP4.1*) using SSR markers in mungbean. The details of the linked makers to these QTLs and QTLs linked to other minor elements are given in Table 1.

Improving the nutritional status of elite breeding lines requires functional information about the genetic network controlling important traits such as Fe, Zn, and protein content, oil content, pro-vitamin A, an array of amino acids, etc. Although impressive progress has been made in the identification of a large number of QTLs of nutritional traits in major food staples, an important challenge is their introgression into the agronomically superior background without compromising the yield and their popularization in malnourished areas.

## Genomic Approaches For Enhancing Nutritional Quality in Major Food Crops

**Figure 1:**
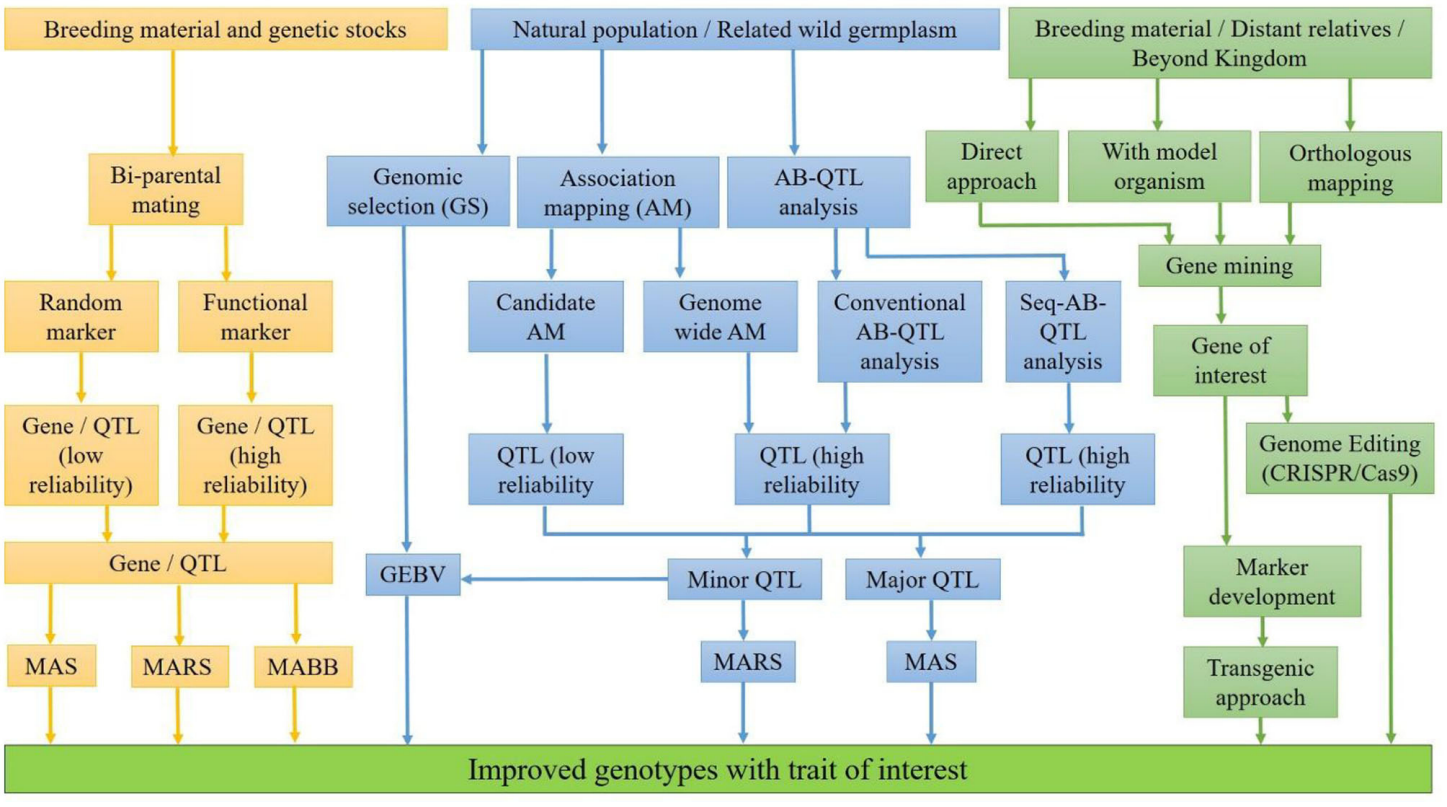
Approaches for developing genotypes with enhanced nutritional traits.

For the development of nutritionally rich cultivars, a suitable breeding strategy, available genetic diversity, and modern genomics information are needed. Sufficient genetic variability for nutritional quality traits has been explored in cultivars, direct progenitors, and wild relatives over the years. Moreover, a big treasurer of genetic diversity across the kingdoms can be explored and utilized through genetic engineering and genome editing. This diversity should be converted into nutritionally rich cultivars. Therefore, proper understanding of the genetic basis of nutritional quality traits and interaction with the environment is of utmost importance for an efficient breeding program. The integration of modern genomics, physiology coupled with precise phenotyping and advance breeding methodologies, has effectively revealed the genes and the metabolic pathways of quality traits. The identification and the incorporation of nutritional quality traits is being facilitated by several approaches (Figure 1), each having its own advantages and disadvantages.

The identification of loci for trait of interest can be facilitated with QTL mapping by the population developed from bi-parental mating or the natural population having large variability for the trait of interest. In bi-parental QTL mapping approach, two parents having a difference for the target trait are crossed to generate a mapping population of the mortal (F2, F3, etc.) or immortal (RILs, NILs, etc.) in nature. QTLs can also be identified with natural population with the help of association mapping (AM), *viz*., genome-wide AM, candidate-gene based AM, and bulk segregant analysis (BSA). In genome-wide AM, polymorphism of the whole genome is considered at once for mapping of locus, while in candidate-gene based AM, the polymorphism of a particular genomic region for mapping of locus is considered. In BSA, target genomic regions linked with trait of interest are identified with simultaneous transfer from wild source into recipient during backcross program. The reliability of the identified QTLs depends on the closeness of the marker with the linked locus/QTLs. QTLs identified with functional markers/genic markers are more reliable because these markers are from the transcribed region and/or part of the identified locus. Therefore, SNP genotyping coupled with precise phenotyping, bioinformatics, and modern statistical algorithm specific to available data has enabled the identification of genomic regions controlling our traits of interest. Once genomic regions/QTLs associated with nutritional traits are identified, the next step is to utilize them for the development of nutritionally rich cultivars. The decision to transfer the target nutrional traits with molecular breeding depends on the relative eliteness and adaptation of the donor parent, the genetic complexity of the trait, the relative cost of phenotypic assays, the trait-linked marker assays, and genome profiling (119). If a nutritional trait is controlled by few loci, in elite genetic background, marker-assisted selection (MAS) is suggested. However, if the donor parents are landraces or distant relatives, marker-assisted backcross breeding (MABB) would be suitable for the transfer of trait. When the nutritional trait is controlled by a large number of genes with low genetic variance, genomic selection would be the choice to transfer the target trait (119). Genomic selection (GS) in crop breeding is gaining prominence due to the current availability of low-cost high-density genotyping with high-density DNA array chips and genotyping by sequencing (120, 121). In GS, all marker effects across the genome are estimated together by fitting the phenotypic and the marker data of the base training population with an appropriate statistical model (120). Then, genomic estimated breeding values (GEBVs) of any unknown genotypes having only marker information are generated with these marker effects. GEBVs predict the breeding values of unobserved genotypes for selection in the testing population (122, 123). With marker-assisted selection, nutritional traits like QPM and pro-vitamin A, controlled by major genes in maize, have been transferred in elite hybrids and released for commercial cultivation in India from ICAR-IARI, Delhi, India. These hybrids, namely, “Pusa Vivek QPM-9 Improved” having high QPM and pro-vitamin A in 2017, “Pusa Vivek Hybrid-27 Improved” having high pro-vitamin A, and “Pusa HQPM5 Improved” and “Pusa HQPM7 Improved,” both having high QPM and pro-vitamin A in 2019, have been released for commercial cultivation in India so far (119, 124). In bread wheat, some success stories, but not of commercial success, of the introgression of major gene Gpc-B1 (high protein content) with MAS have been reported by several researchers (125–129). In durum wheat, one successful example of a cultivar having high protein content using MAS, namely, “Desert King-High Protein,” had been developed at the University of California wheat breeding program (130). However, little success has been achieved in the development of nutritionally rich commercial cultivars with MAS in other food crops. The simple reason is that most of these traits are controlled by a large number of genes having a little effect individually in the expression of these traits. In bread wheat, the major QTLs for Zn content, namely, QZn2A and QZn7B, were each having only 11.9% genetic variance (19); therefore, introgression of these QTLs with MAS into an elite background is very difficult. In addition, genotype by environment interaction increases the complexity many folds in the transfer of target traits. Therefore, for complex quantitative traits governed by many minor QTLs, genomic selection (GS) would be a better choice than MAS (131). As a matter of fact that the genes or major QTLs for nutritional qualities are known, there are large numbers of minor QTL effects on the expression of trait. To support this fact, the betaine aldehyde dehydrogenase (BADH 2) gene solely is not able to explain the variation of aroma in rice because background minor QTLs contribute to the overall fragrance in rice (132, 133). Therefore, integration of minor effect QTLs for nutritional traits can be effectively accomplished by employing the recurrent selection under breeding cycles under GS.

### Genome Editing Technologies for Enhancing Nutritional Quality in Major Food Crops

Although conventional breeding is currently the most preferred and widely used approach for developing nutrient-enriched genotypes/varieties, it is labor-intensive and resource consuming, and it usually takes several years to develop a final product right from the screening of large germplasm lines, making crosses, and selection of desired recombinants that can finally turn into the commercial variety. Genetically modified (GM) crops that have beneficial traits are developed by the transfer of transgene(s) of known function into elite crop varieties. The transgenic approach can be an alternative for the development of biofortified crops when the genetic variation for a particular nutrient is limited or absent in the germplasm. Unlimited genetic variation across the boundary of species can be explored through this approach. However, their use is largely affected by unsubstantiated health and environmental safety concerns raised by non-governmental organizations and many developed countries. Government regulatory frameworks in many developed countries that aim to safeguard human and environmental biosafety have led to significant cost barriers to the rapid widespread adoption of new GM traits (134). As a result, the advantages of GM traits have been restricted to a small number of cultivated crops such as maize (high lysine), soybean (high oleic acid), potato (high amylopectin), cassava (high content of iron, β-carotene, and protein), and canola (phytate degradation) (3). Genome editing technologies facilitate efficient, precise, and targeted modifications at the genomic loci (135). The first-generation genome editing technologies that use ZFNs (136) and TALENs (137) have been around for two decades. They are labor-intensive, time-consuming, and involve a complex procedure to gain target specificity. However, second-generation genome editing techniques like CRISPR/Cas9 system (138) require less time and cost and provide simplicity and ease of targeted gene editing. All of these technologies use typical sequence-specific nucleases that can be induced to recognize specific DNA sequences and to generate double-stranded breaks (DSBs). The DSBs are repaired by plants' endogenous repair mechanisms, *viz*., non-homologous end joining, which can lead to the insertion or deletion of nucleotides, thereby causing gene knockouts, or by homologous recombination, which can cause gene replacements and insertions (139). The risks involved in altering genomes through the use of genome editing technology are significantly lower than those associated with GM crops because most edits alter only a few nucleotides, producing changes that are not unlike those found throughout naturally occurring populations (140). Once the genomic editing agents have segregated out, there is no way to distinguish between a “naturally occurring” mutation and a gene edit. Thus, the introduction of genome editing into modern breeding programs should facilitate rapid and precise crop improvement. Genome editing techniques often produce gene knockout mutants, gene replacement, and insertion mutants, thus becoming a potent tool in the improvement of traits of nutritional quality in major food crops (Table 2).

**Table 2:** Nutritional quality traits improved by genome editing technologies in major food crops.

**Crop species**	**Gene editing technology**	**Target gene**	**Trager trait**	**References**
Wheat	CRISPR/Cas9	α-gliadin gene family	Low gluten content	(141)
Wheat	CRISPR/Cas9	α- and γ-gliadin gene family	Low gluten content	(142)
Rice	CRISPR/Cas9	*SEBIIb*	High amylose content	(143)
Rice	TALENs	*OsBadh2*	Enhanced aroma	(144)
Rice	CRISPR/Cas9	*OsFAD2-1*	Oleic acid content	(145)
Rice	CRISPR/Cas9	*Osor*	Enhanced β-carotene content	(146)
Rice	CRISPR/Cas9	*Rc*	Proanthocyanidinis and anthocunins	(147)
Maize	CRISPR/Cas9	*Wx1*	Waxy corn	(148)
Soybean	TALENs	*FAD2-1A, FAD2-1B*	High oleic acid content	(149)
Soybean	TALENs	*FAD2-1A, FAD2-1B*	High oleic, low linoleic acid content	(150)
Soybean	CRISPR/Cas9	*FAD2-2*	High oleic, low linoleic acid content	(151)
Soybean	TALENs	*GmPDS11* and *GmPDS18*	Carotenoid biosynthesis	(152)
Peanut	CRISPR/Cas9	*ahFAD2*	High oleic acid content	(153)
Potato	CRISPR/Cas9	*Wx1*	High amylopectin	(154)
Potato	TALENs	*VInv*	Low reducing sugars	(155)
Rapeseed	CRISPR/Cas9	*FAD2*	High oleic acid content	(156)
Rapeseed	CRISPR/Cas9	*BnaFAD2*	High oleic acid content	(157)

The first-generation genome editing technique TALENs has been used for modifying and enhancing the nutritional profile of major food crops. Soybean oil contains high levels of polyunsaturated linoleic and linolenic acid, which contribute to oxidative instability. This problem is often addressed through partial hydrogenation. However, partial hydrogenation increases the levels of *trans*-fatty acids, which have been associated with cardiovascular disease. Altering the composition of soybean oil by increasing the level of oleic acid and decreasing the levels of linoleic and linolenic acids may help reduce the need for hydrogenation. Soybean lines with high oleic acid and low linoleic acid contents were generated by introducing mutations in the two fatty acid desaturase 2 genes (FAD2-1A and FAD2-1B) (149) and fatty acid desaturase 3A (*FAD3A*) gene by directly delivering TALENs into *fad2-1a fad2-1b* soybean plants (150) and FAD2-2 gene using CRISPR/Cas9 (151), thus improving the shelf-life and heat stability of soybean oil. The same gene ahFAD2 was mutated for the isolation of high oleic acid lines in peanut using CRISPR/Cas9 (153).

Rice is a staple food crop feeding more than half of the world population. High amylose content and resistant starch improves human health and reduces the risk of serious diseases including hypertension, diabetes, and colon cancer (158). The CRISPR/Cas9 technology has been successfully used to create high-amylose rice by targeting two rice branching enzymes, *SBEI* and *SBEIIb* (143). Rice bran oil (RBO) contains many valuable healthy constituents, including oleic acid. Fatty acid desaturase 2 (FAD2) catalyzes the conversion of oleic acid to linoleic acid in plants. To produce high oleic/low linoleic RBO, Abe et al. (145) disrupted the OsFAD2-1 gene by CRISPR/Cas9-mediated targeted mutagenesis and developed rice lines with high oleic acid (twice that of the wild type) and low linoleic acid content. Enhancing the level of β-carotene is an important target of biofortification in major food crops because it is a precursor of pro-vitamin A. Endo et al. (146) were successful in accumulating β-carotene in rice callus (up to 2.86 ± 1.41 μg/g fresh weight) by identifying the putative ortholog of the cauliflower or gene in rice, *Osor*, and modifying the Osor gene *via* genome editing using CRISPR/Cas9. The majority of the rice varieties grown over the world are having a white pericarp. However, rich diversity also exists for brown, red, or purple/black pericarp. The red pericarp contains high levels of proanthocyanidins and anthocyanins which have been recognized as health-promoting nutrients (159). The red coloration in the grains of wild rice is controlled by two complementary genes, *Rc* and *Rd* (160, 161). Recently, Zhu et al. (147) successfully converted three elite white-pericarp rice varieties into red ones having high proanthocyanidin and anthocyanin content without compromising the yield potential through CRISPR/Cas9-mediated functional recovery of the recessive rc allele. Aromatic rice is popular worldwide for the characteristic fragrance of its grains. More than 100 volatile compounds were detected in the flavor of cooked fragrant rice. The presence of a defective *badh2* allele encoding BADH 2 results in the synthesis of 2-acetyl-1-pyrroline, which is a major fragrance compound in aromatic rice. Shan et al. (144) reported the creation of fragrant rice from a non-fragrant variety via the targeted knockout of *OsBADH2* using the TALEN method.

Using CRISPR/Cas9, DuPont Pioneer, in 2016, developed the first waxy corn hybrid by knocking out the maize waxy gene *Wx1*, making more than 97% amylopectin and essentially eliminating amylose from the kernel (148). The variety with high amylopectin starch content has higher digestibility and many industrial applications. The release of commercial hybrids with this trait is planned for 2020 (148). The researchers from Swedish Agricultural University developed high-amylopectin potatoes by knocking out the granule-bound starch synthase gene using CRISPR/Cas9 (154). The high-amylopectin potato starch has uses in both food and technical applications.

In potato tubers, the low temperature during cold storage stimulates the accumulation of reducing sugars that influence the quality of the product. Upon high-temperature processing, these reducing sugars react with free amino acids, resulting in brown, bitter-tasting products and elevated levels of acrylamide—a potential carcinogen. To minimize the accumulation of reducing sugars, vacuolar invertase (VInv) gene was knocked out by the TALENs technique (155).

The gluten protein of wheat which is responsible for the unique viscoelastic properties of wheat-derived foods also triggers gluten sensitivity in susceptible individuals commonly known as celiac disease. Because of the complexity of the Gli-2 locus and the high copy number of a-gliadin genes, conventional breeding and mutagenesis have failed to develop low-immunogenic wheat varieties for patients with celiac disease. Low-gluten, transgene-free wheat lines with much reduced immunoreactivity, using CRISPR/Cas9, have been developed (141, 142) and will serve as donors for introgressing low gluten trait into elite wheat varieties.

Modification of fatty acid composition is one of the primary objectives in the improvement of oilseed brassicas. The fatty acid desaturase 2 gene, *FAD2*, is a key gene that affects oleic, linoleic, and linolenic acids. The CRISPR/Cas9-mediated genome editing system has been applied for developing lines with high oleic acid (more than 80%) of rapeseed (*Brassica napus*) (156, 157) and in the emerging oilseed plant *Camelina sativa* (162, 163) by modifying the *FAD2* gene. In yet another study in *Camelina*, the content of very long fatty acids was reduced by knocking out the FAE1 gene (164).

Genome editing technologies, because of their efficiency, high specificity, and amenability to multiplexing, have increasingly become popular genomic tools for enhancing the nutritional value of our major food crops. The mutant line generated through CRISPR/Cas9 will provide new genetic diversity for the traits of agronomic and nutritional importance for breeding in an unprecedented way.

### Developing Nutrient-Enriched Varieties Through Conventional and Genomics-Assisted Breeding

Developing nutrient-enriched varieties of food crops through conventional plant breeding is the most preferred approach. The improvement of a particular trait requires the availability of sufficient and useful genetic diversity. This genetic diversity may be present in cultivated genotypes or can be introgressed from wild/related/progenitor species or can be created through directed mutagenesis. Work on the development of nutrient-rich varieties of major food crops is ongoing in various public and private sector organizations with the support of important programs like HarvestPlus, Global Alliance for Improved Nutrition (GAIN), Project ENABLE (Expanding Nutrition Access by Building capacity, Linking Initiative and Enhancing policy), HEALTHGRAIN Forum, etc. The purpose of all these programs is to improve the nutritional status of the food crops and make them available for all the people, especially the most vulnerable ones. Conventional breeding supported by the above-mentioned programs has yielded several nutrient-enriched varieties in important food crops (Table 3).

**Table 3:** Nutrient-enriched important biofortified crops developed through conventional and genomics-assisted breeding.

**Crop**	**Biofortified nutrient**	**Name of variety**	**Country**	**Developing institute**	**References/source**
Wheat	Zinc and iron	WB 02	India	ICAR-IIWBR, India, and CIMMYT	(165)
		HPBW 01	India	PAU, India, and CIMMYT	(166)
	Iron	HD 3171	India	ICAR-IARI, India	(167)
	Zinc	Zinc Shakti	India	CIMMYT	(166)
	Zinc	Zncol 2016	Pakistan	CIMMYT	(166)
	Zinc	BARI Gom 33	Bangladesh	Bangladesh Wheat and Maize Research Institute, and CIMMYT	(166)
	Zinc	BHU 1 BHU 3 (Akshay) BHU 5 BHU 6	India	Banaras Hindu University, CIMMYT, and HarvestPlus	(168)
	Protein content	HD 3226	India	ICAR-IARI, India	(169)
		PBW 757	India	PAU, India	(170)
	Yellow pigment (carotene)	HI 8627 HI 8759 HI 8777	India	ICAR-IARI, India	(170)
	Anthocyanin (colored wheat)	Scorpion	Austria	Crop Research Institute, Prague	(171)
		PS Karkulka	Slovak Republic	National Agricultural and Food Center	(172)
		NABIMG 9 NABIMG 10 NABIMG 11	India	National Agri-Food Biotechnology Institute, New Delhi	(173)
		Indigo	Austria	BOKU-University of Natural Resources and Life Sciences, Austria	(174)
		Black-grained wheat	China	Shanxi University, China	(175)
Rice	Zinc	Jalmagna	India	Landrace collection	(176)
	Iron	IR 72/Zawa Bonday (IR 68144-313-2-2-3)	India, Philippines	International Rice Research Institute, Philippines	(176)
	Zinc	BRRIdhan 62 BRRIdhan 64 BRRIdhan 72	Bangladesh	Bangladesh Rice Research Institute (BARI) and HarvestPlus	(177)
	Zinc	CR Dhan 45	India	National Rice Research Institute, India	(178)
	Protein	CR Dhan 310	India	National Rice Research Institute, India	(178)
Maize: Quality Protein Maize (QPM)	Lysine and tryptophan	Protina (composite) Ratan (composite)	India	GBPUAT, Pantnagar	(179)
		Shakti (composite) Shakti1 Shaktiman 1 Shaktiman 2 Shaktiman 3 Shaktiman 4	India	Indian Institute of Maize Research (IIMR), India	(179)
		HQPM 1 HQPM 5 HQPM 7	India	CCS Haryana Agricultural University (CCSHAU), Hisar, India	(179)
		Vivek QPM 9	India	ICA-VPKAS Almora, India	(179)
		Pusa HM 4 Improved^a^ Pusa HM 8 improved^a^ Pusa HM 9 Improved^a^	India	ICAR-IARI, India	(124)
		QPHM 200 and QPHM 300	Pakistan	National Agricultural Research System and CIMMYT	(180)
		BHQPY 545 BHQP 542 Melkassa 1Q Melkassa 6Q MHQ 138	Ethiopia	Bako Agricultural Research Center, Ethiopia, and CIMMYT	(181–183)
		GH-132-2	Ghana	Agricultural Research Centers Ethiopia and CIMMYT	
		BR-451 (OPV) BR-473 (OPV) Assum Preto (OPV)	Brazil	University of Ghana and CIMMYT	
		HB-PROTICTA	Guatemala	Guatemala's Institute for Agricultural Science and Technology and CIMMYT	
		Obatampa GH Mambia CMS 473 CMS 475 K9101	Guinea	Crop Research Institute, Kumasi and International Institute of Tropical Agriculture (IITA)	
		Obatampa GH	Benin		
		Susuma (OPV)	Mozambique	CIMMYT, Mexico	
		Obatampa Espoir	Burkina Faso	CIMMYT, Mexico	
		Obatampa	Cameroon	CIMMYT, Mexico	
		Obangaina (OPV)	Uganda	CIMMYT, Mexico	
		HQ INTA-993 NB-Nutrinta (OPV)	Nicaragua	CIMMYT, Mexico	
		EV 99 QPM	Nigeria	CIMMYT, Mexico	
		Ev 99 QPM	Togo	CIMMYT, Mexico	
		Lishe-K1	Tanzania	CIMMYT, Mexico	
		EV 99 QPM DMRESR WQPM Susuma	Senegal	CIMMYT, Mexico	
		HQ-31	Honduras	CIMMYT, Mexico	
		HQ-61	El Salvador	CIMMYT, Mexico	
		ICA	Colombia	CIMMYT, Mexico	
		KH 500Q KH 631Q WSQ 104	Kenya	CIMMYT, Mexico	
		Lishe-H1, Lishe-H2	Tanzania	CIMMYT, Mexico	
		ZS 261Q	Zimbabwe	CIMMYT, Mexico	
		Quian 2609 Zhongdan 9409 Zhongdan 3850 Yun Yao 19 Yun You 167 Lu Dan 167 Lu Dan 206 Lu Dan 207 Lu Dan 807 Hybrid 2075	China	Guizhou, China CAAS, China CAAS, China Yunnan, China Yunnan, China Shandong, China Shandong, China Shandong, China Shandong, China Sichuan, China	
		H551C H553C H519C H368EC H369EC	Mexico	CIMMYT, Mexico	
		QS-7705	South Africa	CIMMYT, Mexico	
		FONAIAP	Venezuela	CIMMYT, Mexico	
		INIA	Peru	CIMMYT, Mexico	
		HQ-2000	Vietnam	NMRI, Vietnam	
	QPM+ pro-vitamin A	Pusa Vivek QPM 9 Improved^a^	India	ICAR-IARI, India	(12, 178)
		Pusa HQPM5 Improved^a^ Pusa HQPM7 Improved^a^	India	ICAR-IARI, India	(178, 184)
Maize: orange maize	Pro-vitamin A	Pusa Vivek Hybrid 27 Improved^a^	India	ICAR-IARI, India	(12, 178)
		Sam Vita 4-A, Sam Vita 4-B, Muibaki 3, Muibaki 2, Muibaki 1, GV662	DR Congo	CIMMYT, IITA, and HarvestPlus	(185)
		Ahoɔ*dzin, Dzifoo, AhoO*fε, CSIR-CRI Honampa, CSIR-CRI Odomfo, CSIR-CRI Owanwa	Ghana	CIMMYT, IITA, and HarvestPlus	(185)
		GV671A (HPH1301), GV673A (HPH1303), GV665A (HP1005), GV662A (HP1002), GV664A (HP1004)	Zambia	CIMMYT, IITA, and HarvestPlus	(186)
		Ife maizehyb-3 Ife maizehyb-4 Sammaz 38 (OPV) Sammaz 39 (OPV)	Nigeria	CIMMYT, IITA, and HarvestPlus	(185)
		Nafama, Abebe, Duba, Kodialan, Dakan	Mali	CIMMYT, IITA, and HarvestPlus	(185)
		HPH1317, HP1005	Tanzania	CIMMYT, IITA, and HarvestPlus	(185)
		ZS242 (HP1005), ZS244 (HPH1301), ZS246 (HPH1302), ZS248 (HPH1303)	Zimbabwe	CIMMYT, IITA, and HarvestPlus	(185)
		MH39A, MH40A, MH42A, MH43A	Malawi	CIMMYT, IITA, and HarvestPlus	(185)
Maize: zinc maize	Zinc	BIO-MZn01	Colombia	CIMMYT and HarvestPlus	(187)
Pearl millet	Iron and zinc	Dhanshakti Hybrid ICMH 1201	India	ICRISAT and HarvestPlus	(188)
	Iron and zinc	HHB 299	India	CCSHAU, Hisar and ICRISAT	(178)
	Iron and zinc	HHB 311	India	CCSHAU, Hisar and ICRISAT	(188)
	Iron	AHB 1200	India	Vasantrao Naik Marathwada Krishi Vidyapeeth, Parbhani and ICRISAT	(178)
	Iron and zinc	RHB 233	India	SKN Agricultural University, Rajasthan and ICRISAT	(188)
Sorghum	Iron and zinc	ICSR 1401 (Parbhani Shakti)	India	VNMKV Agricultural University Parbhani and ICRISAT	(189)
	Ion	SAMSORG 45 SAMSORG 46	Nigeria	Nigerian National Agricultural Research System and ICRISAT	(190)
Common bean	High iron	NAROBEAN 1 NAROBEAN 2 NAROBEAN 3 NAROBEAN 4C NAROBEAN 5C	Uganda	HarvestPlus and International Center for Tropical Agriculture (CIAT)	(191)
	High iron	CAB 2, RWR 2145, RWR 2245, MAC 42, MAC 44, RWV 1129, RWV 2887, RWV 3006, RWV 3316, and RWV 3317	Rwanda	HarvestPlus and International Center for Tropical Agriculture (CIAT)	(192)
	High Iron	HM 21-7, RWR 2245, PAV 1438, Namulenga, Cod MLV 059, and Cuarentino	Democratic Republic of Congo	HarvestPlus and International Center for Tropical Agriculture (CIAT)	(193, 194)
Lentil	Iron and zinc	Pusa Ageti Masoor (L4717) L4704 Pusa Vaibhav	India	ICAR-IARI, New Delhi and ICARDA	(178, 195)
		Barimasur 4 Barimasur 5 Barimasur 6 Barimasur 7 Barimasur 8	Bangladesh	Bangladesh Agricultural Research Council (BARI) and ICARDA	(196, 197)
		Shekhar Khajuraho 1 Khajuraho 2 Sisir Shital Khajuraho Masuro 3 (RL 4)	Nepal	Nepal Agricultural Research Council and ICARDA	(198)
		Alemaya	Ethiopia	ICARDA	(199)
		Idlib 2, Idlib 3, Idlib 4	Syria	ICARDA	
		Myveci-2001	Turkey	ICARDA	
		Beleza	Portugal	ICARDA	
Soybean	Kuntiz trypsin inhibitor (KTI)-free	NRC 127^a^	India	ICAR-Indian institute of Soybean Research, India	(200)
		UEL 175^a^	Brazil	Sate University of Londrina, Parana, Brazil	(201)
	KTI and lipoxygenase 2 free	NRC 142^a^	India	ICAR-Indian institute of Soybean Research, India	(202)

a *Varieties/hybrids developed through genomics-assisted breeding*.

#### Wheat

Wheat is a staple cereal crop worldwide and one of the most preferred food crops for biofortification. The major target in wheat biofortification is the improvement of iron (Fe) and zinc (Zn) content in the grain. Wheat is blessed with a wider genetic variation for these two traits contributed by wild/progenitor species. This rich genetic diversity introgressed from *Triticum durum*-, *Triticum spelta*-, and *Triticum dicoccum*-based synthetic hexaploid wheat (SHW) into cultivated bread wheat led to the development of four high-Zn varieties in India and Pakistan. In India, the variety “Zinc Shakti” (Chitra), with 14 ppm Zn, was developed through participatory variety selection and further registered by private seed companies and growers. This variety has profitable yield potential and matures nearly 2 weeks earlier than common wheat. Two more varieties, *viz*., “WB 02” and “HPBW 01,” were developed in 2017 by ICAR-India Institute of Wheat and Barley Research (ICAR-IIWBR) and Punjab Agricultural University (PAU), Ludhiana, respectively, for cultivation in the North-Western Plain Zone (NWPZ) of India (166) (Table 3). Wheat variety HD 3171, developed by ICAR-Indian Agricultural Research Institute (ICAR-IARI), New Delhi, is shown to have high iron content (47.2 ppm) (167). In Pakistan, the variety Zncol 2016 with +9 ppm Zn has been released for commercial cultivation (166) (Table 3). Apart from SHW, variation for high zinc content is present in wheat germplasm and varieties. In 2018, Bangladesh has witnessed the release of the first biofortified blast-resistant variety “Bari Gom 33″ with +7 ppm Zn advantage, developed with the support of International Maize and Wheat Improvement Center (CIMMYT). In 2012, the HarvestPlus program of CGIAR, in alliance with Banaras Hindu University, India, and CIMMYT, developed high-Zn genotypes designated as BHU 1, BHU 3 (Akshay), BHU 5, and BHU 6. Through public–private partnership, HarvestPlus has reached more than 50,000 wheat farmers in the Eastern Gangetic Plain of India (168) (Table 3). Improvement in protein content is also an important target in wheat breeding. In general, protein content is believed to be negatively correlated with grain yield. However, in 2018, ICAR-Indian Agricultural Research Institute (IARI) New Delhi and PAU Ludhiana become successful in developing high-protein (~13%) and high-yielding bread varieties HD 3226 (169) and PBW 757, respectively, for NWPZ of India (170). Improvement of β-carotene content is a major target in durum wheat breeding program. The presence of yellow pigment in the grain generally measured as the Yellow Pigment Index (YPI), which is also a measure of β-carotene content, is an important quality trait and indicates an antioxidant in durum wheat. The bright yellow color of pasta due to the presence of yellow pigment in the flour is an indicator of good pasta quality. In India, durum wheat varieties such as WH 896, PDW 233, HI 8759, and HI 8777 with YPI >19 are developed and commercialized (170). In a wheat breeding program, one of the significant aims is to develop non-traditional genotypes having an added value which would impart a relevant health benefit. This biofortified colored wheat with high anthocyanin content is an extensive area of research. Anthocyanin can act as an antioxidant that removes harmful free radicals from the body and helps in the prevention of heart diseases, diabetes, obesity, and cancer. A winter wheat variety “Scorpion,” with blue grain color, was registered in 2011 in Austria and in 2012 in Europe (171). Another winter wheat variety with purple grain “PS Karkulka” was developed by the National Agricultural and Food Center, Slovak Republic, in 2014 (172). In India, anthocyanin-rich wheat genotypes, *viz*., NABIMG 9, NABIMG 10, and NABIMG 11, with enhanced Zn content have been developed and registered (173) (Table 3).

#### Rice

The majority of the Asian population prefers rice as their staple food. This milled and polished rice is a poor source of some minerals and proteins; therefore, it becomes a preferred crop for biofortification. Gregorio et al. (176) screened improved cultivars, new plant type lines, local landraces, and lines from wild/related species for Fe and Zn content. It was reported that Fe (15.6 mg/kg) and Zn (378 mg/kg) content was higher in wild/related species. They also reported that an Indian floating rice local cultivar “Jalmagna” had 40% more Zn concentration than the mega variety IR 64. An improved line (IR 68144-313-2-2-3) with high grain Fe concentration (21 ppm in brown rice) was developed by the International Rice Research Institute. This line was derived from a cross of IR 72 and “Zawa Bonday,” a traditional variety from India (176). In 2015, Bangladesh Rice Research Institute (BARI) has released their third high-Zn rice variety “BRRI dhan 72” with the support of HarvestPlus (177) (Table 3). Two earlier varieties, *viz*., BRRI dhan 62 (2013) and BRRI dhan 64 (2014), were also enriched with Zn. The Indian Council of Agricultural Research (ICAR) has one of the mandates to improve the nutritional quality in high-yielding varieties of cereals, pulses, oilseeds, vegetables, and fruit crops. In rice, India has released two biofortified varieties of rice, *viz*., “CR Dhan 310” having high protein content (10.3% in polished rice), which was developed by the National Rice Research Institute, Cuttack, and “DRR Dhan 45,” having high Zn content (22.6 ppm) in polished grain, developed by the Indian Institute of Rice Research Hyderabad (178) (Table 3).

#### Maize

Maize is the third most important food grain, following wheat and rice. In recent years, the pro-vitamin A-enriched maize hybrids/varieties developed across the globe tell a success story of the biofortification program. In India, maize hybrid Pusa Vivek Hybrid Improved, enriched in pro-vitamin A, has been released for commercial cultivation (12). In Zimbabwe, orange maize rich in pro-vitamin A is bred especially for human consumption. The CIMMYT with HarvestPlus has also released biofortified orange maize variety “ZS 422” for commercial cultivation in Zimbabwe. Similarly, in many African countries where malnutrition is a serious concern, biofortified orange maize varieties have been released (185, 186) (Table 3). Maize has a significant limitation; it lacks two essential amino acids, lysine and tryptophan, which are not synthesized in the human body. Maize breeders E. Villegas and SK Vasal at CIMMYT had developed quality protein maize (QPM) genotypes with high lysine and tryptophan by incorporating “opaque 2” gene along with genetic modifiers. In recent years, India has released a good number of QPM hybrids for commercial cultivation which includes Ratan, Protina, Shakti, Shakti 1, Shaktiman 1, Shaktiman 2, Shaktiman 3, Shaktiman 4, Shaktiman 5, HQPM 1, HQPM 5, HQPM 7, Vivel QPM 9, Vivek QPM 21, and Pratap QPM hybrid 1 with enhanced endospermic content of lysine and tryptophan (179) (Table 3). In addition to this, new QPM hybrids, *viz*., Pusa HM 4 Improved, Pusa HM 8 improved, and Pusa HM 9 Improved, with 0.68–1.06% lysine and 2.97–4.18% tryptophan were released by IARI, New Delhi, in 2017 (124) (Table 3). India released its first pro-vitamin A-enriched QPM hybrid “Pusa Vivek QPM 9 Improved” with high pro-vitamin A (8.5 ppm), lysine (2.76 ppm), and tryptophan (0.74%) (178). In the year 2020, IARI, New Delhi, again came up with three more hybrids, *viz*., Pusa HQPM 5 Improved and Pusa HQPM 7 Improved, having high pro-vitamin A in the QPM hybrid background, and one hybrid Pusa Vivek Hybrid 27 Improved, having high pro-vitamin A (200) (Table 3). The Global Maize Program of CIMMYT is the frontrunner in developing and releasing QPM hybrids for commercial cultivation in developing countries. The CIMMYT has also released QPM hybrids in Pakistan (QPHM 200 and QPHM 300), Ethiopia (BHQPY 545 and BHQP 542), Ghana (GH-132-28), Guatemala (HB-PROTICTA), Nicaragua (HQ INTA-993), Honduras (HQ-31), El Salvador (HQ-61), Colombia (ICA), Kenya (KH 500Q, KH 631Q), Tanzania (Lishe-H1, Lishe-H2), Ethiopia (Melkassa 1Q, Melkassa 6Q, MHQ 138), Zimbabwe (ZS 261Q), and China (Quian 2609, Zhong Dan 9409) (Table 3). Thus, QPM can be a better alternative to eliminate malnutrition in developing countries such as African nations where maize is a staple diet. In 2018, CIMMYT has released a new zinc-enriched maize variety, BIO-MZn01, in Colombia to help combat malnutrition in South America. This variety contains 36% more zinc on average than other maize varieties, with grain yield of 6–8 t/ha (187). Furthermore, QPM combined with high Fe and Zn, if developed, will be a favorable choice to tackle the problem of malnutrition.

#### Pearl Millet

Anemia is a significant health concern in millions of women and children in developing countries, especially in African and Asian countries. The reason for this is their staple diet which is deficient in iron (Fe). The pearl millet variety “Shakti” officially released in African country Niger and developed by International Crop Research Institute for Semi-Arid and Tropics (ICRISAT) has higher Fe (65 mg/kg) and Zn (icrisat.org). In India, high-Fe (75 mg/kg) and high-Zn (40 mg/kg) variety “Dhanashakti” and hybrid “ICMH 1201” have been released by ICRISAT during 2012–2014 (Table 3). Recently, ICRISAT, in collaboration with state agricultural universities in India, has developed and released four biofortified hybrids, *viz*., AHB 1200 (iron 73.0 ppm), HHB 299 (iron 73.0 ppm; zinc 41.0 ppm), and HHB 311 and RHB 233 with high Fe and Zn content (178) (Table 3). These biofortified varieties and hybrids have 7.5 to 8.0 mg of iron and 3.5 to 4.5 mg zinc per 100 gm of pearl millet. It is estimated that consumption of 200 g/day biofortified pearl millet-based food will provide 70% of dietary Fe and Zn requirement in men and women, and 130 g/day will give 100% requirement for children (188). With these improvements, pearl millet is becoming ‘Smart Food Crop’ on account of its high Fe and Zn content, improved levels of drought, heat, and salinity tolerance, high protein with balanced amino acids, high dietary fibers, and gluten-free protein.

#### Sorghum

Sorghum is the grain of the twenty first century for Africa. It is the only viable food grain for millions of food-insecure people. In Nigeria, the Nigerian National Agricultural Research System and ICRISAT have released two sorghum varieties, “SAMSORG 45″ and “SAMSORG 46,” with 129 ppm Fe content. These biofortified varieties are higher yielding and resistant to Striga. In India, during 2018, ICRISAT has developed sorghum variety ICSR 14001 (later named as Parbhani Shakti), with 45 ppm Fe and 32 ppm Zn. Besides these, it has low phytate content (4.14 mg/100 g), which will increase the bioavailability of nutrients (Table 3).

#### Common Bean

Common bean is important for nutrient and poverty alleviation in the developing countries of Central America, Andean regions of South America, and Eastern and Southern Africa (203). Common bean has wide variation for Fe content, i.e., up to 110 ppm, which is much higher than the target of 40 ppm set by HarvestPlus. Therefore, developing Fe-rich varieties through breeding is quite easy. The HarvestPlus and International Center for Tropical Agriculture (CIAT) have released five varieties (NAROBEAN 1, NAROBEAN 2, NAROBEAN 3, NAROBEAN 4C, and NAROBEAN 5C) in Uganda, 10 varieties (CAB 2, RWR 2145, RWR 2245, MAC 42, MAC 44, RWV 1129, RWV 2887, RWV 3006, RWV 3316, and RWV 3317) in Rwanda, and six varieties (HM 21-7, RWR 2245, PAV 1438, Namulenga, Cod MLV 059, and Cuarentino) in the Democratic Republic of Congo (191–194) (Table 3).

#### Lentil

Lentil is a highly nutritious pulse crop, well-adapted to dry land, in poor-soil-fertility areas of Africa, Middle-East, Indian-Sub-Continent, Southern Europe, America, Australia, and West Asia (204). Lentil has been a choice in priority for biofortification of mainly Fe, Zn, and Se. The International Center for Agricultural Research in Dry Areas (ICARDA) is working with national programs in India, Bangladesh, and Nepal to breed Fe- and Zn-enriched lentil varieties. In recent years, ICARDA's research partnership with Bangladesh Agricultural Research Council (BARI) led to the release of five Fe- and Zn-enriched lentil varieties, namely, Barimasur 4, Barimasur 5, Barimasur 6, Barimasur 7, and Barimasur 8 (196, 197) (Table 3). These biofortified lentil varieties have reached 8,20,000 farmers in Bangladesh. In Nepal, five varieties (Shekhar, Khajuraho 1, Khajuraho 2, Sisir, and Shital) with 81–98 ppm Fe and >54 ppm Zn are rapidly gaining acceptance and popularity among 4,00,000 farmers (198). In India, three biofortified Fe-rich lentil varieties “Pusa Vaibhav” (102 ppm), IPL 220 (73–114 ppm), and L 4704 are becoming popular among the farmers of North Eastern India (178, 195) (Table 3).

#### Soybean

Elimination of Kunitz trypsin inhibitor (KTI) from soybean seeds is one of the important breeding objectives. For its inactivation, preheating of soy flour is required before using it in food and feed products. The heat treatment not only increases the processing but also affects the seed protein quality and solubility (205). For the development of KTI-free soybean lines, MABB approach was adopted to introgress the kti allele (source PI542044) in the genetic background of popular varieties in India: DS9712, DS9814(204), and JS97-52 (206). At ICAR–Indian Institute of Soybean Research, India, a soybean variety NRC 142, free from KTI and lipoxygenase 2, has been developed through marker-assisted breeding (https://iisrindore.icar.gov.in/pdfdoc/NRC142News.pdf). In Brazil, a KTI-free variety UEL 175 has also been developed through marker-assisted breeding (201).

## Conventional and Genomic-Assisted Breeding: Advantages and Limitations

Biofortification is targeted mainly to the rural poor in developing countries who are mainly dependent on staple foods for meeting their energy and nutrient demand and have very remote access to nutrient-rich fortified foods. The design of conventional plant breeding programs capitalizes on the available variability in the germplasm to breed nutrient-rich crops. The breeding for biofortification has been successful as several varieties have been released in different crops and are in cultivation in India, Pakistan, Zambia, etc. Both conventional and genomics-assisted breeding programs capitalize on the existing variability in the germplasm to breed nutrient-rich crops. The main advantage of both breeding strategies is that the varieties developed through these approaches have no yield penalty from an economic point of view and are also equally competitive to the earlier varieties in cultivation. The acceptability of varieties developed does not have an issue involved as in the case of genetically modified crops. Catering the nutrients through biofortification will uplift the livelihood as well as the nutritional status of the target population as this system is cost-effective and highly sustainable. Most of the quality traits are quantitative in nature and are highly influenced by environmental factors, because of which the progress of breeding varieties will be slow through conventional plant breeding. In many cases, the primary gene pool has limited variability; then, transferring the target trait from wild relatives poses serious constraints with respect to time duration and linked unfavorable genes (linkage drag). A large number of QTLs linked to key nutrient traits have been identified in different crops, which are amenable for marker-assisted selection, but in crops having a large genome size, like wheat, finding closely linked genes to the target traits was challenging, but with the advent of novel genomic tools, like genome-wide association mapping, genomic selection and also whole genome sequence data finding markers tightly linked to the trait of interest are possible. Marker-assisted selection can be applied to transfer the genes/QTLs once closely linked markers are available. Numerous studies have been reported in literature regarding mapping of QTLs linked to different biofortification traits. By using genomics-assisted breeding, the time duration for the development of biofortified varieties can be reduced to 5 to 6 years as compared to conventional breeding, and the problem of linkage drag is also addressed in genomics-assisted breeding while transferring the target trait from wild relatives.

However, both of these approaches have some limitations; for example, if the target micronutrient does not have variability in the crop germplasm, then genetic improvement is impossible. In some cases, it would be impossible to breed for a specific trait using conventional means, and the timescale and the effort involved may be quite unrealistic, e.g., improving Se concentration in wheat grains (207). Rice plants possess the whole pathway to synthesize β-carotene; this pathway is fully active in leaves but turned off in the grain. By adding only two genes, a plant phytoene synthase (psy) and a bacterial phytoene desaturase (crt I), the pathway is turned back on and β-carotene consequently accumulates in the grain. On the other hand, it is not possible either through conventional or genomics-assisted breeding to switch on the pathway. In marker-assisted breeding, the linkage between the trait and the marker determines the success of a biofortification program. In some cases, the genetic background can alter the expression of the transferred traits while transferring QTLs. In case of cross-pollinated crops, the biofortified hybrids may lose the trait in one or few generations of inbreeding, so the farmers have to buy a fresh hybrid seed to maintain the required trait. In most of the developing countries, there is no demarcation or segregated procurement with respect to normal or biofortified wheat crop after harvest. So, all wheats get equal treatment with respect to the procurement price. If there is a scope for the segregated procurement of biofortified crops to be linked with some monetary benefit, such will encourage the adoptability of biofortified crops. Given the widespread prevalence of micronutrient deficiencies, conventional breeding coupled with the integration of genomic tools will be effective in providing access to micronutrient-rich food to the target deficient population in developing countries as they capitalize on staple food crops for the delivery of micronutrients.

## Perspectives of Enhancing Nutrients With Conventional and Genomics-Assisted Breeding

Increasing the content of nutritional elements/minerals through genetic biofortification has been demonstrated in major food crops, and several varieties have been released (Table 3). The traits governing nutritional contents are mostly polygenic and involved GX E interaction; hence, improvement through conventional plant breeding is possible, provided that the time required for the release of the variety is generally long. This is a sustainable and cost-effective solution for the elimination of malnutrition. Advancements in molecular genetics have aided in precise mapping and dissecting the molecular basis of biofortification. Several major-effect and stable QTLs for biofortification traits, such as GPC, minerals, and pigments, have been identified in different crops (Table 1). These QTLs can be transferred into elite lines using marker-assisted selection, provided that the markers are tightly linked to the trait of interest, which is also sometimes challenging in crops having a large genome size. The advent of genomic selection has led to drastically reduced cost and increased throughput of genotyping assays, in combination with advances in high-throughput phenotyping. Through genomic selection, several QTLs pertaining to nutritional traits have been mapped and combined into elite lines in wheat (20). Increasing the micronutrients through genetic and genomic approaches is well-demonstrated where genetic variation exists in the germplasm. However, if genetic variation is not available in the germplasm, then novel techniques of genome editing (Table 2) and genetic engineering need to be employed, as demonstrated by establishing the pro-vitamin A pathway in golden rice (208). Golden rice has proved to elevate the pro-vitamin A level in rice, but due to strict GMO regulations and unexpected health risks, transgenic crops still have not found acceptance. Otherwise, golden rice has ample to offer to the world's malnutrition-prone population.

The other less explored nutritional elements, like low sugar and omega 3- fatty acids, are gaining importance with the upsurge of chronic illnesses like diabetes and cardiovascular diseases. The challenge is to alter the starch structure of rice to lower its digestibility so that it provides a slow and steady supply of energy but without sacrificing its cooking and eating quality. A low-glycemic-index (GI) rice variety with hard texture is not preferred in the Southeast Asian market, where soft and sticky types are the benchmarks of rice quality. Variations in rice germplasm for low GI along with hard and cohesive texture have been assessed using genome-wide association mapping and are currently being used as pre-breeding materials to develop low-GI lines targeted toward certain market segments, particularly in Asia (209). Omega-3 fatty acids have proven to be very essential for human health due to their multiple health benefits like skin and hair growth as well as for proper visual, neural, and reproductive functions of the body, but the concern is that they are not produced by the human body. The requirement of these fatty acids is generally met by deep sea fishes as variation in plants is not that much. The entire DHA biosynthetic pathway was reconstituted in oilseed crop *Brassica juncea* by stepwise metabolic engineering. Transgenic plants produced up to 25% arachidonic acid and 15% eicosapentaenoic acid (EPA), as well as up to 1.5% docosahexaenoic acid (DHA) in seeds (210). However, the accumulation of EPA and DHA was low in transgenic lines, and therefore it was difficult to commercialize. An emerging oilseed crop, *Camelina sativa* seed, contains >30% alpha-linolenic acid, which is the starting substrate required for the synthesis of EPA and DHA, making it a good platform for assembling the ω-3-LC-PUFA synthesis pathway. The co-expression of five genes (OtD6 desaturase from the eukaryotic microalgae *Ostreococcus tauri*, TcD5 desaturase from marine fungus *Thraustochytrium* sp, Piw-3 desaturase from *Phytophthora infestans*, PsD12 desaturase from *Phytophthora sojae*, and D6 fatty acid carbon chain elongation enzyme PSE1 from *Physcomitrella patens*) has demonstrated EPA accumulation of up to 31% in camelina oil (211). The contents of EPA and DHA in this transgenic camelina were comparable to the levels found in fish oil, representing the successful assembly of biosynthetic pathways in commercial oil crop seeds, but there are regulatory issues in the case of transgenics. Genome-wide analysis tools have revealed several genomic regions associated with fatty acid composition in seed oil that had never before been implicated in lipid metabolism (212). By mining sequence data, the candidate enzymes for target fatty acids were identified and subsequently used to manipulate *C. sativa* oil composition toward a superior biofuel and bio-based lubricant oil (213, 214).

In view of this, conventional and genomics-assisted breeding both provide a way of improving the micronutrient status of crops as they have no acceptance problem like the transgenics. An additional advantage that these approaches offer is that farmers can use and retain the seed for food as well as for the next crop plantation. Staple crop biofortification will offer the advantage of delivering the much-needed micronutrients through seeds directly to the malnuorished population in the developing world. Additionally, further studies are needed to improve the bioavailability of these elevated protein, mineral, and pigment concentrations, and the target micronutrients for each crop need to be set by a nutrition specialist in consultation with breeders. The most important issue is making the farmers and populations aware about the nutritional benefits of consuming biofortified foods for their acceptability among the masses. With the immense potential and scope of biofortification, it can be envisaged that this technology can breed nutrient-dense crops that will be crucial in addressing the problem of malnutrition.

## Conclusion

Making our staple food crops nutrient-rich either by enriching our soils with nutrients or making nutrient-rich crops by modern breeding and biotechnological tools such as discovery and mapping of QTLs and their use in marker-assisted breeding will certainly help in eliminating malnutrition to a larger extent. The advanced QTL mapping tools like GWAS and the annotated whole-genome sequence of wheat and other cereals offer new opportunities to study the exact nature of allelic variation and explore the underlying genetic basis and putative gene(s) associated with quality traits. In conclusion, the compiled information on the QTLs on different nutrients flanked by linked marker systems, *viz*., SSR, SNP, DArT, and CAPS, would help in their precise introgression into elite breeding lines/cultivated varieties through MAS in biofortification breeding programs. Impressive work has been done in the identification of QTLs pertaining to nutritional quality and the release of nutritionally rich varieties through conventional breeding in major food crops. However, new avenues in genome editing research are making impressive findings in enriching our food crops to make them nutritionally rich. Genome editing technologies that can rapidly modify genomes in a precise way and will directly enrich the nutritional status of elite varieties could hold a bright future to address the challenge of malnutrition.

## Author Contributions

KG prepared the outline of the contents and coordinated the drafting of the manuscript. KG, SR, MK, VG, PB, and RY wrote the manuscript. NB assisted in final editing and addressing the comments. All authors contributed to the edits and the review.

## Consent For Publication

The manuscript has been approved by all authors.

## Conflict of Interest

The authors declare that the research was conducted in the absence of any commercial or financial relationships that could be construed as a potential conflict of interest.
